# Quinolines-Based SARS-CoV-2 3CLpro and RdRp Inhibitors and Spike-RBD-ACE2 Inhibitor for Drug-Repurposing Against COVID-19: An *in silico* Analysis

**DOI:** 10.3389/fmicb.2020.01796

**Published:** 2020-07-23

**Authors:** Rajaiah Alexpandi, Joelma Freire De Mesquita, Shunmugiah Karutha Pandian, Arumugam Veera Ravi

**Affiliations:** ^1^Department of Biotechnology, School of Biological Sciences, Science Campus, Alagappa University, Karaikudi, India; ^2^Laboratory of Bioinformatics and Computational Biology, Department of Genetics and Molecular Biology, Federal University of Rio de Janeiro State (UNIRIO), Rio de Janeiro, Brazil

**Keywords:** drug repurposing, SARS-CoV-2, main-protease (3CLpro), RNA-dependent RNA-polymerase, spike-ACE2 complex, quinoline based-drugs

## Abstract

The novel coronavirus SARS-CoV-2 disease “COVID-19” emerged in China and rapidly spread to other countries; due to its rapid worldwide spread, the WHO has declared this as a global emergency. As there is no specific treatment prescribed to treat COVID-19, the seeking of suitable therapeutics among existing drugs seems valuable. The structure availability of coronavirus macromolecules has encouraged the finding of conceivable anti-SARS-CoV-2 therapeutics through *in silico* analysis. The results reveal that quinoline,1,2,3,4-tetrahydro-1-[(2-phenylcyclopropyl)sulfonyl]-trans-(8CI) and saquinavir strongly interact with the active site (Cys-His catalytic dyad), thereby are predicted to hinder the activity of SARS-CoV-2 3CLpro. Out of 113 quinoline-drugs, elvitegravir and oxolinic acid are able to interact with the NTP entry-channel and thus interfere with the RNA-directed 5′-3′ polymerase activity of SARS-CoV-2 RdRp. The bioactivity-prediction results also validate the outcome of the docking study. Moreover, as SARS-CoV-2 Spike-glycoprotein uses human ACE2-receptor for viral entry, targeting the Spike-RBD-ACE2 has been viewed as a promising strategy to control the infection. The result shows rilapladib is the only quinoline that can interrupt the Spike-RBD-ACE2 complex. In conclusion, owing to their ability to target functional macromolecules of SARS-CoV-2, along with positive ADMET properties, quinoline,1,2,3,4-tetrahydro-1-[(2-phenylcyclopropyl)sulfonyl]-trans-(8CI), saquinavir, elvitegravir, oxolinic acid, and rilapladib are suggested for the treatment of COVID-19.

## Introduction

The current outbreak of coronavirus disease 2019 (COVID-19), caused by the severe acute syndrome coronavirus-2 (SARS-CoV-2), has been considered as a major anxiety of the twenty first century (Dey et al., 2020). As of May 07, 2020, WHO states that over 3,672,238 cases have been authoritatively affirmed, including 254,045 deaths around the globe. The pathognomonic symptoms of COVID-19 are fever, dry cough, shortness of breath, and dyspnea (Wu et al., 2020). In severe conditions, it causes hypercytokinemia, lymphopenia, disseminated intravascular coagulation, severe acute respiratory syndrome, kidney failure, and eventually death (Thomas-Rüddel et al., 2020). In general, SARS-CoV-2 is a positive sense, long (30,000 bp), single-strand RNA coronavirus that belongs to the family *Coronaviridae* and genus *Betacoronavirus*, which is highly similar to SARS-CoV (Zou et al., 2020). No specific medication for COVID-19 is accessible at the present time. Thus, researchers are seriously searching for suitable vaccines and therapeutic-drugs against COVID-19 (Yao et al., 2020). The fact is that discovery, as well as marketing, of new drugs frequently takes months to years (Stebbing et al., 2020), and hence looking for appropriate therapeutics among existing-drugs seems to be a promising strategy to control the current pandemic of COVID-19 in this critical time.

In the SARS-CoV-2 macromolecules, the large-polyproteins-encoded cysteine protease, called 3-chymotrypsin like protease [3CLpro or main protease (Mpro)], are essential for the viral life-cycle of novel coronavirus (Zhang H. et al., 2020). This enzyme plays a crucial role in the processing of viral polyproteins, which are indispensable for viral maturation and their infectivity (Khan et al., 2020). Subsequently, RNA-dependent RNA polymerase (RdRp) is a key enzyme essential for the viral replication of SARS-CoV-2 (Gao et al., 2020). Due to their crucial roles, these viral proteins are considered as imperative targets for developing antiviral compounds against COVID-19 (Wu et al., 2020). Recently, Choy et al. (2020) reported that the combination of HIV-protease inhibitors such as lopinavir/ritonavir effectively kills SARS-CoV-2 at the cellular level. Similarly, Wang M. et al. (2020) reported that the nucleotide analog RdRp-inhibitor, remdesivir, successfully inhibited SARS-CoV-2 *in vitro*. Hall and Ji (2020) reported zanamivir, indinavir, saquinavir, and remdesivir as SARS-CoV-2 3CLpro inhibitors using *in silico* analysis. Further, Elfiky (2020) also suggested ribavirin, remdesivir, sofosbuvir, galidesivir, and tenofovir as potent drugs against SARS-CoV-2 through docking analysis.

On the other hand, human angiotensin-converting enzyme 2 (ACE-2), a type-I integral membrane protein, has been considered to be the specific and functional receptor for the spike glycoprotein of SARS-CoV-2 (Patel et al., 2014). It is also well-known to play the main role in the rennin-angiotensin system (RAS), which is associated with the regulation of heart function and blood pressure hemeostasis (Oudit et al., 2003). The coronavirus entry into host cells is mediated by the spike glycoprotein, which is a surface transmembrane protein in SARS-CoV-2 (Zhao et al., 2020). The analysis of the receptor-binding motif (RDM) in the Spike glycoprotein revealed that most of the aminoacid residues essential for receptor-binding with ACE-2 were conserved between SARS-CoV-2 and SARS-CoV, demonstrating that these viruses use the same host receptor for cell entry (Yan et al., 2020). Hoffmann et al. (2020) proved that anti-human ACE-2 antibody (R&D Systems, Catalog #AF933) can inhibit the Spike protein-associated entry into cultured cells *in vitro*. Accordingly, human ACE2 is considered as a host target for the treatment of COVID-19 to avoid SARS-CoV-2 from entering host cells (Zhang L. et al., 2020).

The existing quinoline-based antimalarial drugs, hydroxychloroquine and chloroquine, have shown their potential in the treatment of COVID-19 (Kaur et al., 2010), which inspired us to identify the quinoline-based potent inhibitors against the therapeutic targets of SARS-CoV-2 using an *in silico* approach. Due to the drug-like properties and therapeutic potential, quinoline-derived compounds have sustained attention for developing novel drugs in future medicine (O'donnell et al., 2010). Quinolines are nitrogen-containing heterocyclic aromatic compounds, known to be versatile compounds because of their extensive uses in medicine, organic chemistry, and industrial chemistry (Prajapati et al., 2014). They are frequently found in several medicinal plants and are known to have antimalarial, anticancer, antibacterial, anti-fungal, anticonvulsant, anti-inflammatory, anthelminitc, cardiotonic, and analgesic activity (Hussaini, 2016). Some of the compounds with quinoline core are the preferred choice for the treatment of diverse ailments, especially cancer and malaria (Touret and de Lamballerie, 2020).

## Materials and Methods

### Ligand Preparation

Numerous medicinal plants and their phytocompounds have demonstrated their antiviral properties against a large group of viruses. Consequently, the phytocompounds of *Diplocyclos palmatus* leaf extract were subjected to docking analysis in the current study. In previous studies, the tropical medicinal plant of *D. palmatus* has been reported for its anti-biofilm, anti-infection, and anti-photoaging activity using *Caenorhabditis elegans* model (Alexpandi et al., 2019). The list of quinoline-drugs (total 113) was retrieved from DrugBank database (https://www.drugbank.ca/categories/DBCAT000788). The canonical SMILES of the compounds was retrieved from the PubChem database. The canonical SMILES of quinoline,1,2,3,4-tetrahydro-1-[(2-phenylcyclopropyl)sulfonyl]-trans-(8CI) was retrieved from the Guidechem database (https://www.guidechem.com/reference/dic-395649.html). Then, the PDB-format 3D-structure of compounds was downloaded from the Openbabel online server http://www.cheminfo.org/Chemistry/Cheminformatics/FormatConverter/index.html.

### Protein Preparation

The 3D crystal protein-structures of SARS-CoV-2 3CLpro (PDB ID: 6LU7) (Hall and Ji, 2020), SARS-CoV-2 spike protein-ACE-2 receptor-binding domain (RBD) (PDB ID: 6M17) (Wu et al., 2020), and human ACE2 (PDB ID: 1R4L) (Joshi et al., 2020) were obtained from the RCSB PDB database (http://www.rcsb.org/pdb). The 3D crystal structures of SARS-CoV-2 RdRp generated through homology modeling using ICM 3.7.3 modeling software was gifted by Prof. Hua Li, Hubei Key Laboratory of Natural Medicinal Chemistry and Resource Evaluation, School of Pharmacy, Tongji Medical College, Huazhong University of Science and Technology, Wuhan, China (Wu et al., 2020). The energy minimization of targeted protein structures was performed using the YASARA server. The protein preparation was done with AutoDock Tools Version 1.5.6.

### Molecular Docking

The virtual screening of best scoring compounds was performed using the iGEMDOCK with blind-mode docking. The iGEMDOCK tool is a graphical-automatic drug design system mainly used for structure-based virtual screening of drug molecules (Hsu et al., 2011). After the selection of best binding compounds, the interaction on the active domains of therapeutic targets (3CLpro, RdRp, and Spike-ACE2 complex) of selected compounds were analyzed using the AutoDock Vina tool. It is an open-source docking software, which extensively improves the average accuracy of the binding mode predictions of compounds better than other docking tools (Trott and Olson, 2010). It implements a competent optimization algorithm for estimating the affinity of protein-ligand interactions and predicting the plausible binding modes of compounds (Goodsell et al., 1996). Then, the ligand-protein interactions were visualized by Maestro 10 (Schrödinger) (Balasubramaniam et al., 2019). In the present study, to compare the selected quinolines with already reported anti-SARS-CoV-2 drugs, lopinavir (Hall and Ji, 2020) and remdesivir triphosphate (Gordon et al., 2020) were selected as positive inhibitors of SARS-CoV-2 for *in silico* analysis in the present study. Further, the selected quinolines and remdesivir triphosphate were compared with the parental nucleotides (NTPs) of SARS-CoV-2 RdRp for understanding the inhibition mode of viral replication.

### *In silico* Drug-Likeliness and Bioactivity Prediction

The drug likeliness and bioactivity of quinolines were analyzed using the Molinspiration server (http://www.molinspiration.com). Molinspiration tool is a cheminformatics software that provides molecular properties as well as bioactivity prediction of compounds (Mabkhot et al., 2016). In the Molinspiration-based drug-likeness analysis, there are two important factors, including the lipophilicity level (log *P*) and polar surface area (PSA) directly associated with the pharmacokinetic properties (PK) of the compounds (Beetge et al., 2000). In the Molinspiration-based bioactivity analysis, the calculation of the bioactivity score of compounds toward GPCR ligands, ion channel modulators, kinase inhibitors, nuclear receptor ligands, protease inhibitors, and other enzyme targets were analyzed by sophisticated Bayesian statistics (Mabkhot et al., 2016). This was done as the protein families, such as G protein-coupled receptors (GPCR), ion channels, kinases, nuclear hormone receptors, proteases, and other enzymes (RdRp), are the major drug targets of most of the drugs (Hauser et al., 2017).

### *In silico* ADMET Analysis

The PK properties, such as Absorption, Distribution, Metabolism, Excretion, and Toxicity (ADMET), of quinolines were predicted using the admerSAR v2.0 server (http://lmmd.ecust.edu.cn/admetsar2/). The admerSAR server is an open-source computational tool for prediction of ADMET properties of compounds, which makes it a practical platform for drug discovery and other pharmacological research (Guan et al., 2019). In the ADMET analysis, the absorption (A) of good drugs depends on factors such as membrane permeability [designated by colon cancer cell line (Caco-2)], human intestinal absorption (HIA), and the status of either P-glycoprotein substrate or inhibitor. The distribution (D) of drugs mainly depends on the ability to cross the blood-brain barrier (BBB). The metabolism (M) of drugs is calculated by the CYP, MATE1, and OATP1B1-OATP1B3 models. Excretion (E) of the drugs is estimated based on the renal OCT substrate. Then, the toxicity (T) of the drugs is predicted on the Human Ether-a-go-go- related gene inhibition, carcinogenic status, mutagenic status, and acute oral toxicity (Shen et al., 2012).

## Results

### Screening of Potent SARS-CoV-2 3CLpro Inhibitors

**Figure 1:**
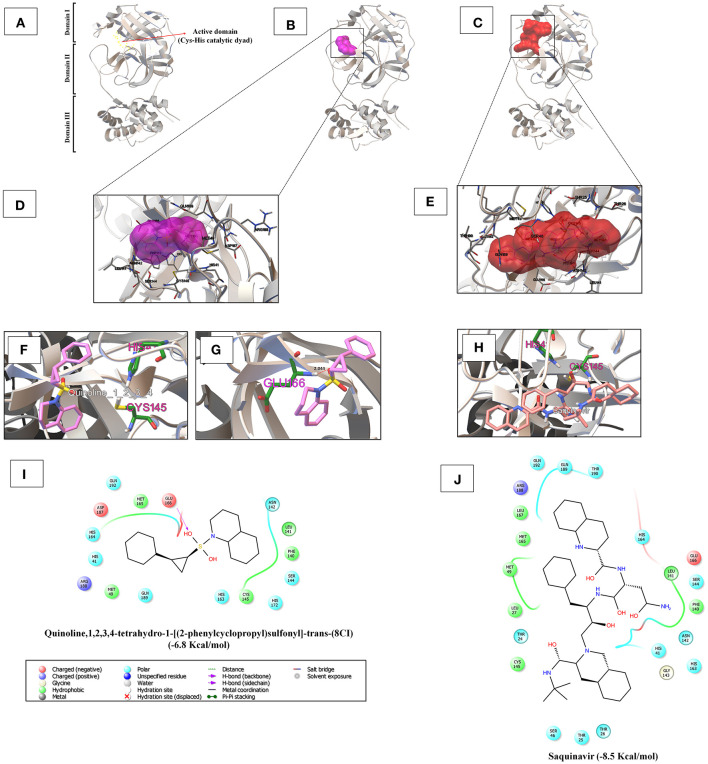
**(A)** The 3CLpro monomer has three domains, namely domain I (residues 8–101), domain II (residues 102–184), and domain III (residues 201–303). The red arrow mark indicates the region of the active site of 3CLpro, which is located in the gap between domains I and II, and has a CysHis catalytic dyad (Cys145 and His41). **(B,C)** Depicts the binding region of the quinoline,1,2,3,4-tetrahydro-1-[(2-phenylcyclopropyl)sulfonyl]-trans-(8CI) (−6.8 Kcal/mol) and saquinavir (−8.5 Kcal/mol) with SARS-CoV-2 3CLpro, respectively. **(D,E)** Shows the super-position view of the binding sites and their interacting amino acids of quinoline,1,2,3,4-tetrahydro-1-[(2-phenylcyclopropyl)sulfonyl]-trans-(8CI) and saquinavir with SARS-CoV-2 3CLpro, respectively. **(F,H)** Indicates the interaction with the active catalytic dyad of SARS-CoV-2 3CLpro (Cys145 and His41). **(G)** Hydrogen bond formation of quinoline,1,2,3,4-tetrahydro-1-[(2-phenylcyclopropyl)sulfonyl]-trans-(8CI) with Glu166 residues of 3CLpro at 2.044 Å distance. **(I,J)** Reveal the interacted aminoacid residues of SARS-CoV-2 3CLpro with quinoline,1,2,3,4-tetrahydro-1-[(2-phenylcyclopropyl)sulfonyl]-trans-(8CI) and saquinavir.

In Table S1, the iGEMDOCK-based virtual screening result reveals that among the 17 phytocompounds, the novel quinoline (NQ) identified from *D. palmatus*, quinoline,1,2,3,4-tetrahydro-1-[(2-phenylcyclopropyl)sulfonyl]-trans-(8CI), has a lower binding energy (-6.8 Kcal/mol) with SARS-CoV-2 3CLpro, compared to other phytocompounds. As shown in Figures 1D,I, the NQ builds five hydrophobic interactions (Cys145, Met49, Met165, Phe140, and Leu141), 8 polar interactions (His41, His164, Gln192, Gln189, His63, His172, Ser144, and Asn142), two negative-charged interactions (Asp187 and Glu166), and one unspecified residue interaction (Arg188). As shown in Figure 1G, the NQ forms a hydrogen bonding interaction with Glu166 (2.044 Å distance). Importantly, the NQ has effectively interacted with the active site of catalytic-dyad (Cys145 and His41) of the SARS-CoV-2 3CLpro (Figures 1B,F), and hence we assumed that the NQ is able to hinder the protease activity of SARS-CoV-2 like lopinavir (−6.6 Kcal/mol) (Figure S1), which is a protease-inhibitor based anti-SARS-CoV-2 drug reported for COVID-19 (Choy et al., 2020).

**Figure 2:**
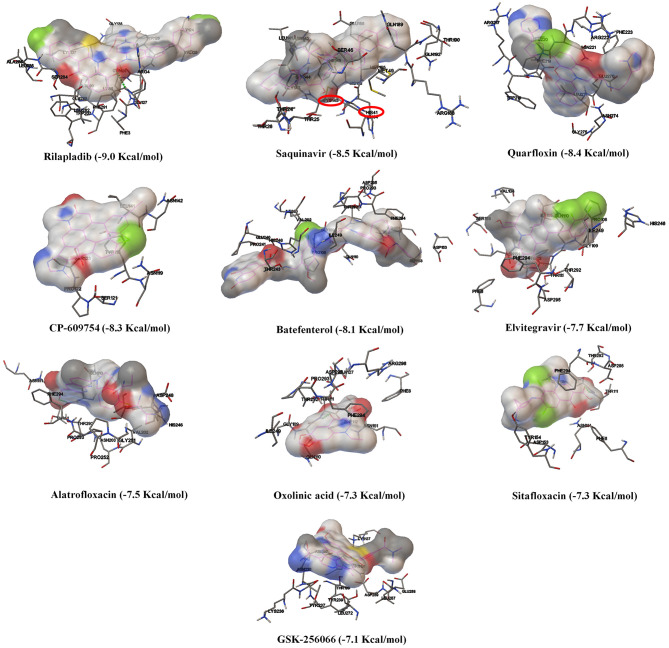
The binding patterns and aminoacid interactions of the best scoring quinolines with SARS-CoV-2 3CLpro. The red circle-marking indicates the interaction with the active site of SARS-CoV-2 3CLpro (CysHis catalytic dyad).

Based on the virtual screening results of SARS-CoV-2 3CLpro, rilapladib, saquinavir, oxolinic acid, elvitegravir, batefenterol, sitafloxacin, CP-609754, GSK-256066, alatrofloxacin, and quarfloxin were predicted to be the best compounds from the commercial quinoline-based drugs (Figure 2 and Table S2). Out of these compounds, only saquinavir (−8.5 Kcal/mol) was found to interact with the active catalytic-domain (Cys145 and His41) of the SARS-CoV-2 2CLpro (Figures 1H,C). As shown in Figures 1E,J, saquinavir forms seven hydrophobic interactions (Cys145, Met165, Met49, Leu27, Leu167, Leu141, and Phe140), 12 polar interactions (His41, Asn142, His163, Ser144, Ser46, Thr25, Thr26, Thr24, Gln192, Gln189, Thr190, and His164), one negative-charged interaction (Glu166), and one glycine interaction (Gly143) with SARS-CoV-2 3CLpro. Therefore, for the development of strong interactions at the CysHis catalytic dyad, saquinavir was predicted to obstruct the 3CLpro activity.

### Screening of Potent SARS-CoV-2 RdRp Inhibitors

**Figure 3:**
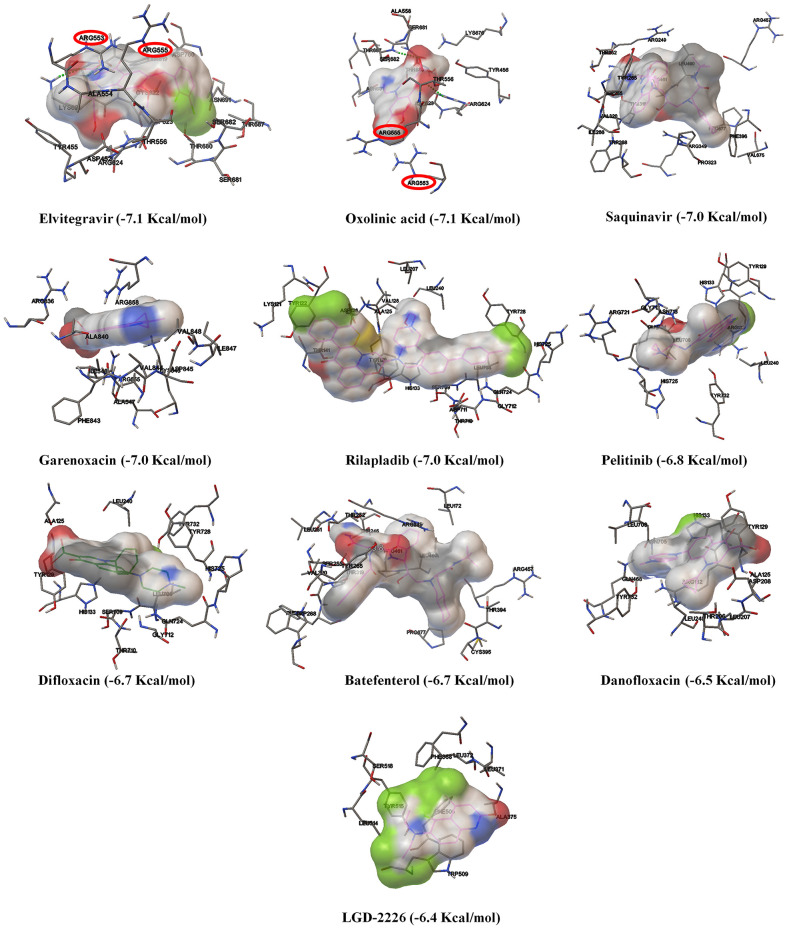
The binding patterns and aminoacid interactions of the best scoring quinolines with SARS-CoV-2 RdRp. The red circle-marking indicates the interaction with the NTP entry channel of SARS-CoV-2.

**Figure 4:**
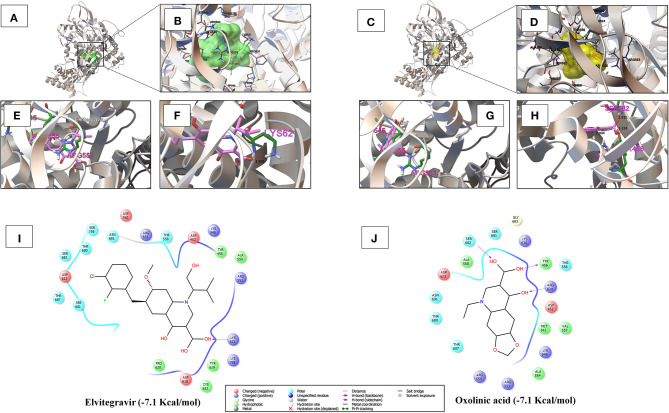
**(A,B)** Shows the binding region and close-up view of the interacting amino acids of elvitegravir (−7.1 Kcal/mol) with SARS-CoV-2 RdRp, respectively. **(C,D)** Individually depicts the binding region and super-position view of the interacting amino acids of oxolinic acid (−7.1 Kcal/mol) with SARS-CoV-2 RdRp, respectively. **(E,G)** Visualizes the interaction with the NTP entry channel of SARS-CoV-2 RdRp (a set of hydrophilic residues such as Lys545, Arg553, and Arg555) of elvitegravir and oxolinic acid. **(F,H)** Shows the hydrogen bond formation of elvitegravir (Lys621 with a distance of 1.931 Å) and oxolinic acid (Thy456, Ser682, and Arg624 with a distance of 1.224, 1.815, and 2.904 Å, respectively). **(I,J)** Illustrate the interacted aminoacid residues of SARS-CoV-2 3CLpro with elvitegravir and oxolinic acid.

As shown in Figure 3 and Table S3, amongst the tested-quinolines, elvitegravir, oxolinic acid, saquinavir, garenoxacin, rilapladib, pelitinib, difloxacin, batefenterol, danofloxacin, and LGD-2226 were shown to be the best-docked compounds against SARS-CoV-2 RdRp. Figures 4A,C reveals that elvitegravir and oxolinic acid have the same binding energy (−7.1 Kcal/mol) and same binding site at SARS-CoV-2 RdRp, which is almost similar to natural nucleotides such as ATP (−7.6 Kcal/mol), UTP (−7.1 Kcal/mol), GTP (−7.7 Kcal/mol), and CTP (−7.1 Kcal/mol) (Figure S2). As shown in Figures 4B,I, elvitegravir forms five hydrophobic interactions (Tyr455, Ala554, Tyr619, Pro620, and Cys622), seven polar interactions (Thr556, Thr680, Ser681, Ser682, Thr687, Asn691, and Ser759), four negative-charged interactions (Asp452, Asp618, Asp623, and Asp760), and five unspecified residue interactions (Lys545, Arg553, Arg555, Lys621, and Lys798) with SARS-CoV-2 RdRp. Notably, elvitegravir builds a hydrogen bond interaction with Lys621 residue (1.931 Å distance), as shown in Figure 4F. Similarly, it formed five hydrophobic interactions (Tyr456, Met542, Ala554, Val557, and Ala558), six polar interactions (Thr556, Thr680, Ser681, Ser682, Thr687, and Asn691), two negative-charged interactions (Asp452 and Asp623), and five unspecified residue interactions (Lys545, Arg553, Arg555, Arg624, and Lys676) (Figures 4D,J). Subsequently, oxolinic acid forms three hydrogen bonding interactions with Thy456 (1.224Å), Ser682 (1.815Å), and Arg624 (2.904) residues of the SARS-CoV-2 RdRp (Figure 4H). As shown in Figures 4E,G, both elvitegravir and oxolinic acid have the ability to bind with the NTP binding channel (a set of hydrophilic residues such as Lys545, Arg553, and Arg555) of the SARS-CoV-2 RdRp, similar to remdesivir triphosphate (−7.8 Kcal/mol) (Figure S3), which is a RdRp-inhibitor based anti-SARS-CoV-2 agent reported for COVID-19 (Gao et al., 2020; Gordon et al., 2020). As shown in Figure S4, due to the same binding sites, we expect these two quinolines (elvitegravir and oxolinic acid) can readily interact with the NTP binding channel of SARS-CoV-2 RdRp, more quickly than the parental-nucleotides (NTPs) such as ATP, UTP, GTP, and CTP. Hence, these two quinoline drugs are anticipated to arrest the viral replication of SARS-CoV-2, as is seen with remdesivir triphosphate.

### Screening of Potent Inhibitor for Spike Protein-RBD-ACE2 Interaction

**Figure 5:**
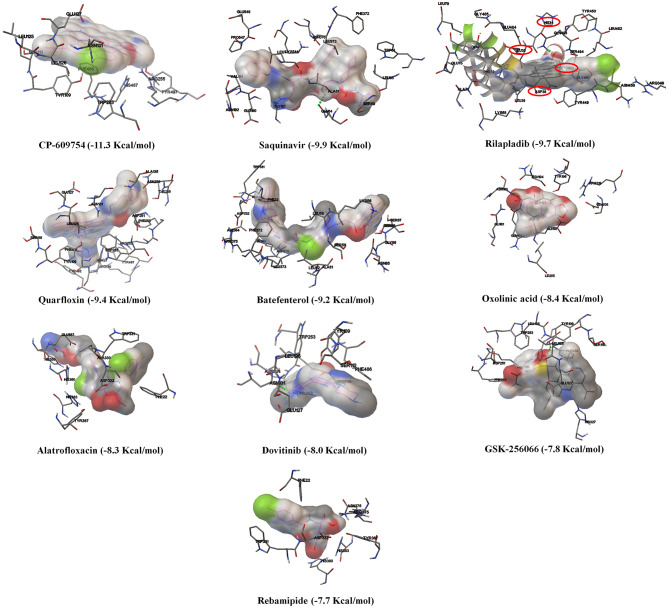
The binding patterns and aminoacid interactions of the top ten scoring quinolines with the RBD interface of the Spike-ACE2 complex. The red circle-marking indicates the interaction with the RBD interface of Spike-ACE2 complex.

**Figure 6:**
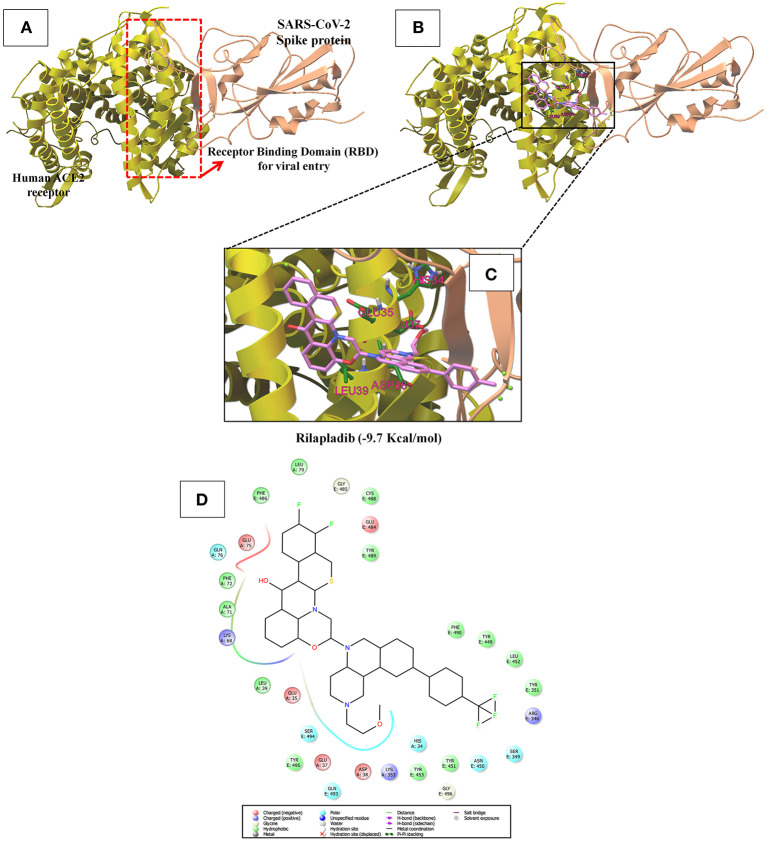
**(A)** Shows the receptor binding domain (RBD) of SARS-CoV-2 Spike protein with human ACE-2 receptor for viral entry into host cells. **(B)** The interrupting mode of rilapladib (−9.7 Kcal/mol) on the interface of RBD of the Spike protein-ACE2 complex. **(C)** Super-position view of the binding interations of rilapladib on the interface of RBD of the Spike protein-ACE2 complex. **(D)** Shows the interacted aminoacid residues of rilapladib on the interface of RBD of the Spike protein-ACE2 complex.

In Figure S5 and Figure 6A, the crystal structure of spike protein-ACE2 (PDB: 6M17) revealed that the aminoacid residues of ACE2, including Gln24, Thr27, Asp30, Lys31, His34, Glu35, Glu37, Asp38, Tyr41, Gln42, Met82, Lys353, Gly354, Asp355, and Arg357, were recognized as the Spike protein receptor-binding domain (RBD) or entry receptor site to invade the target cells (Benítez-Cardoza and Vique-Sánchez, 2020). Based on the virtual screening results of ACE2 protein, CP-609754, saquinavir, rilapladib, quarfloxin, batefenterol, oxolinic acid, alatrofloxacin, dovitinib, GSK-256066, and rebamipide were predicted as the best compounds, exhibiting a high binding affinity to ACE2 with low energy (Figure 5 and Table S4). However, these quinolines were not predicted to interact with the RBD of the Spike-ACE2 complex (Figure 5). The only compound that could target the RBD interface between Spike and ACE2 was rilapladib, as shown in Figure 6B. Rilapladib was predicted to be positioned on the central shallow pit of the RBD of the Spike-ACE2 complex with strong interactions (Figure 6D). The aminoacid residues of the Spike-RBD-ACE2 complex that interact with rilapladib were His34, Glu35, Glu37, Asp38, and Leu39, as shown in Figure 6C. As a result of superimposing the Spike-RBD-ACE2 complex to the rilapladib-RBD-ACE2 complex, an individual overlap of rilapladib with the interface of ACE2 was observed, signifying that rilapladib may possibly interrupt the interaction of the Spike-RBD-ACE2 complex.

### *In silico* Prediction of Drug-Likeness Property and Bioactivity Score

The adequacy of therapeutic drugs mainly depends on the molecular property and bioactivity of the compounds (Shen et al., 2012). To predict the drug-likeness and bioactivity of the selected quinolines, the *in silico* molecular property assessment was performed using the Molinspiration tool. This tool measures the _mi_log*P* value (Octanol-water partition coefficient log*P*) and TPSA (Topological polar surface area) values of the compounds using Bayesian statistics. The result shows that the _mi_log*P* value of quinoline,1,2,3,4-tetrahydro-1-[(2-phenylcyclopropyl)sulfonyl]-trans-(8CI) (3.63), saquinavir (4.26), elvitegravir (3.58), and oxolinic acid (0.68) were predicted as having ideal lipophilicity (log*P* < 5) (Han et al., 2019); rilapladib (7.33) was predicted as having poor lipophilicity (log*P* > 5) in the aspect of absorption and permeation (Figure S6). The TPSA of the quinoline,1,2,3,4-tetrahydro-1-[(2-phenylcyclopropyl)sulfonyl]-trans-(8CI) (37.38), elvitegravir (88.77), oxolinic acid (77.77), and rilapladib (54.79) were < 100, showing that these compounds had superior oral-absorption or membrane permeability than saquinavir (166.75), lopinavir (119.99), and remdesivir triphosphate (289.53) (Bakht et al., 2010). On the other hand, the majority of drug targets of existing drugs are in one of the following protein families: G protein-coupled receptors (GPCR), ion channels, kinases, nuclear hormone receptors, proteases, and other enzymes. In Figure S7, the *in silico* bioactivity prediction analysis also divulges that the NQ and saquinavir were predicted as protease inhibitors. Subsequently, elvitegravir and oxolinic acid were predicted as enzyme inhibitors, which means that these are able to inhibit other enzymes, including RdRp enyzme, except G protein-coupled receptors (GPCR), ion channels, kinases, nuclear hormone receptors, and proteases. This is because the protein families are the major drug targets of most of the drugs (Hauser et al., 2017). These data support the outcome of the predicted *in-silico* activity of selected quinolines against SARS-CoV-2.

The half-life (t_1/2_) of drugs is a valuable pharmacokinetic factor as it provides an exact indication of the duration of time that the effect of the drug continues in an individual. The period of action of a drug is called its half-life. The distribution half-life (t_1/2a_) is the time required to divide the plasma concentration by two after reaching pseudo-equilibrium, and not the time needed to eliminate half the administered dose. The elimination half-life (t_1/2b_) of drugs is defined as the time required for the concentration of the drug to reach half of its original value in the plasma or the total amount in the body. As mentioned in Table 1, the elimination half-life (t_1/2b_) of saquinavir (670.84 g/mol) is 7–12 h and their elimination half-life is 9–15 h (Taylor et al., 2001). The distribution half-life (t_1/2a_) of elvitegravir is calculated at 8.7 h, and the elimination half-life (t_1/2b_) as 12.9 h (Cada et al., 2013). Oxolinic acid (261.23 g/mol) has 1.3 and 84 h as its distribution half-life and elimination half-life, respectively (Samuelsen et al., 2003). Unfortunately, the half-life of rilapladib (735.81 g/mol) as well as quinoline,1,2,3,4-tetrahydro-1-[(2-phenylcyclopropyl)sulfonyl]-, trans-(8CI) (313.41 g/mol) are not available in the database. On the other hand, the elimination half-life of SARS-CoV-2 protease inhibitor lopinavir (628.8 g/mol) measured at 6.9 ± 2.2 h. Similarly, the reported anti-COVID-19 drug remdesivir has a very short half-life (0.39 h) and hence the human esterases hastily converted the remdesivir into nucleoside triphosphate metabolite (remdesivir triphosphate). However, the produced remdesivir triphosphate has a longer half-life of ~20 h in humans, in which the metabolite acts as an NTP analog and slows down the viral replication of SARS-CoV-2 (Eastman et al., 2020). Due to the long half-life of the NTP analog of remdesivir triphosphate, only one dose is required for daily administration for treating COVID-19. The data obtained revealed that the selected quinolines are significantly long in terms of drug half-life, in which the quinolines treatment will be successful in the drug repurposing against COVID-19.

**Table 1:** Half-life, molecular weight, and pharmacological functions of the suggested quinoline drugs for drug-repurposing against COVID-19.

**S. No**.	**Drug/Compound Name**	**DrugBank ID**	**Structure**	**Molecular weight (g/mol)**	**Half-life (h)**		**Pharmacological function**	**Binding energy with the therapeutic targets of COVID-19**	**Drug-repurposing for COVID-19**
					**Distribution half-life (t_**1/2a**_)**	**Elimination half-life (t_**1/2b**_)**			
1	Quinoline,1,2,3,4-tetrahydro-1-[(2-phenylcyclopropyl)sulfonyl]-, trans- (8CI)	Not available	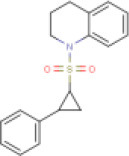	313.41	Not available	Not available	• Protease Inhibitor	−6.8 Kcal/mol with 3CLpro	In this study
2	Saquinavir	DB01232 (APRD00623)	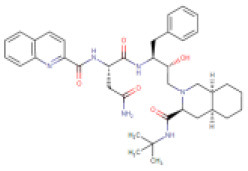	670.84	7–12	9–15	• Anti-HIV Agent (Kim et al., 1998) • Anti-Infective Agent (Noble and Faulds, 1996) • Protease Inhibitor (Kim et al., 1998)	−8.5 Kcal/mol with 3CLpro	In this study
3	Lopinavir (Reported anti-SARS-CoV-2 agent)	DB01601 (EXPT00388)	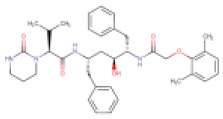	628.8	Not available	6.9 ± 2.2	• Experimental Unapproved Treatment for COVID-19 (Choy et al., 2020) • Anti-HIV Agent (Walmsley et al., 2002) • Protease Inhibitor (Agarwal et al., 2007)	−6.6 Kcal/mol with 3CLpro	Choy et al., 2020
4	Elvitegravir	DB09101 (DB05618)	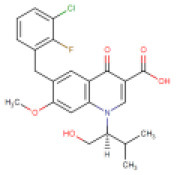	447.88	8.7	12.9	• Anti-HIV Agent (Ramanathan et al., 2011) • Anti-viral for Systemic Use (Lampiris, 2012) • Enzyme Inhibitor (Shimura et al., 2008)	−7.1 Kcal/mol with RdRp	In this study
5	Oxolinic acid	DB13627	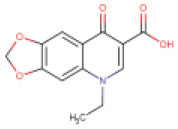	261.23	1.3	84	• Anti-bacterial Agent (Barry et al., 1984) • Anti-Infective Agent for Urinary Infections (Irgi et al., 2015) • Enzyme Inhibitor (Wright et al., 1981)	−7.1 Kcal/mol with RdRp	In this study
6	Remdesivir triphosphate (Reported anti-SARS-CoV-2 agent)	DB14761	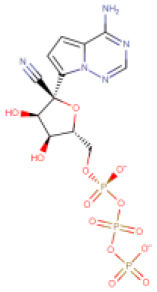	527.17	Not available	20	• Experimental Unapproved Treatment for COVID-19 (Gordon et al., 2020; Wang Y. et al., 2020)	−7.8 Kcal/mol with RdRp	Gordon et al., 2020; Wang Y. et al., 2020
7	Rilapladib	DB05119 (DB05256)	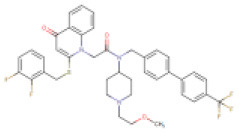	735.81	Not available	Not available	• Anti-Alzheimer's Disease (Husna Ibrahim et al., 2020) • Lp-PLA2 inhibitor (Shaddinger et al., 2014)	−9.7 Kcal/mol with Spike protein-ACE2 complex	In this study

### *In silico* ADMET Analysis

In Table 2, the absorption (A) analysis reveals that the NQ and oxolinic acid were predicted to have high Caco-2 permeability. A HIA of <30% is classified to be poorly absorbed. The result reveals that all the quinolines were predicted to be highly absorbed by the human intestine. P-glycoprotein, a member of ATP-binding trans-membrane glycoprotein, excretes incoming drugs or other chemicals from the cells (Äänismaa and Seelig, 2007). The results revealed that the NQ and oxolinic acid are non-substrates or non-inhibitors for P-glycoprotein and other compounds are substrates/inhibitors for P-glycoprotein. In the distribution (D) analysis, the BBB permeability, logBBB >0.3, is thought to cross the BBB easily (Han et al., 2019). Here, all the quinolines were predicted to be BBB+ and they can easily cross the BBB. In the metabolism (M) analysis, the two main sub-types of cytochrome P450s (CYP) are CYP2D6 and CYP3A4, which are essential enzyme-systems for drug metabolism in the liver (Sams et al., 2004). The results showed that all the quinolines were non-inhibitors to CYP2D6; only NQ and saquinavir were substrates for CYP2D6 and CYP3A4. This data suggested that these quinolines may possibly metabolize in the liver. OATP1B1 and OATP1B3 are transporters expressed on the sinusoidal-membrane of hepatocytes, which interact with therapeutic-drugs as their substrates or inhibitors, and cause clinically relevant drug-drug interactions (Shitara, 2010). The result found that all the selected quinolines were predicted as inhibitors for OATP1B1-OATP1B3. The excretion (E) of drugs is associated with their hydrophilicity and molecular weight. In the kidney, organic cation transporters (OCTs) and multidrug and toxin extrusion proteins (MATEs) are the foremost transporters for the clearance of cationic drugs into the urine (Motohashi and Inui, 2013). The results showed that the quinolines were predicted as non-inhibitors to MATE-1, OCT-1, and OCT-2, which indicates the safety elimination profile of the quinolines. In the toxicity (T) analysis, the result showed that all quinolines were non-carcinogen and non-eye corrosive. In conclusion, the predicted results indicate that the ADMET properties of the quinolines are almost similar to the reported anti-SARS-CoV-2 drugs (lopinavir and remdesivir triphosphate), which promotes repurposing of these quinoline drugs for the treatment of COVID-19.

**Table 2:** Predicted ADMET properties of the selected quinolines.

**ADME parameters**	**Novel quinoline**	**Saquinavir**	**Elvitegravir**	**Oxolinic acid**	**Rilapladib**	**Lopinavir**	**Remdesivir triphosphate**
**Absorption**							
Caco-2 permeability	+	–	–	+	+	+	–
Human Intestinal Absorption (% absorbed)	+	+	+	+	+	+	+
	88.96%	98.13%	96.44%	93.75%	92.54%	96.24%	93.17%
P-glycoprotein inhibitor	–	+	+	–	+	+	+
P-glycoprotein substrate	–	+	+	–	+	+	+
**Distribution**							
Blood Brain Barrier	+	+	+	+	+	+	+
Subcellular localization	Plasma membrane	Mitochondria	Lysosomes	Mitochondria	Mitochondria	Mitochondria	Lysosomes
**Metabolism**							
CYP2D6 inhibition	–	–	–	–	–	–	–
CYP2D6 substrate	+	+	–	–	–	–	–
CYP3A4 inhibition	+	+	–	–	–	–	–
CYP3A4 substrate	–	+	+	–	+	+	+
OATP1B1 inhibitor	+	+	+	+	+	+	+
OATP1B3 inhibitor	+	+	+	+	+	+	+
**Excretion**							
OCT1 inhibitor	–	–	–	–	–	–	–
OCT2 inhibitor	–	–	–	–	–	–	–
MATE1 inhibitor	–	–	–	–	–	–	–
**Toxicity**							
Carcinogens	–	–	–	–	–	–	–
Acute-toxicity (Class)	III	III	III	III	III	III	III
Eye corrosion	–	–	–	–	–	–	–
Eye irritation	–	–	–	+	–	–	–
Human either-a-go-go inhibition	+	+	–	–	+	+	–

*The predicted properties are color-coded to enable easy classification among the quinolines. The color codes are: red for toxic or inhibitor, orange for weak inhibitor or slightly toxic, green for safe or non-inhibitor. The symbols include + for yes and – for no*.

## Discussion

As significant functional biological-macromolecules of coronavirus, the viral protease (3CLpro) and RNA-dependent RNA-polymerase (RdRp) are indispensable for proteolytic processing of the polyproteins as well as viral replication and have been considered as promising drug targets in the treatment of viral diseases (Zumla et al., 2016). Several drugs, including hydroxychloroquine, chloroquine, arbidol, remdesivir, favipiravir, lopinavir/ritonavir, interferon-α, and ribavirin are undergoing clinical trials to assess their anti-viral efficacy and safety level in the treatment of COVID-19 (Dong et al., 2020). Most of the reported anti-SARS-CoV-2 drugs are protease-inhibitors or RdRp-inhibitors (Elfiky, 2020; Wu et al., 2020).

One of the most-characterized therapeutic targets among coronaviruses is inhibiting the 3CLpro activity since this enzyme is crucial for processing the polyproteins that are translated from the RNA molecules (Ghosh et al., 2007; Khan et al., 2020). The 3CLpro, also called Nsp5 (non-structural protein 5), is first routinely cleaved from polyproteins to produce mature enzymes, and subsequently further cleaves downstream Nsps at 11 cleavage sites to release Nsp4-Nsp16 (Wu et al., 2020). 3CLpro directly mediates the maturation of Nsps, which is fundamental in the life-cycle of SARS-CoV-2 (Zhang H. et al., 2020). The 3CLpro monomer has three domains, namely domain I (residues 8–101), domain II (residues 102–184), and domain III (residues 201–303), and a long loop (residues 185–200) links domains II and III, as shown in Figure 1A. The active site of 3CLpro is located in the gap between domains I and II, and has a CysHis catalytic dyad (Cys145 and His41) (Jin et al., 2019; Wu et al., 2020). The active site of SARS-CoV-2 3CLpro is located in the gap between domains I and II, and has a CysHis catalytic dyad (Cys145 and His41) (Muralidharan et al., 2020). The cleavage by 3CLpro arises at the glutamine residue in the P1 position of the substrate through the CysHis catalytic dyad, wherein cysteine thiol functions as the nucleophile in the proteolytic process (Chen et al., 2005). Hence, inhibiting the activity of this enzyme would arrest the viral replication of SARS-CoV-2.

We saw that quinoline,1,2,3,4-tetrahydro-1-[(2-phenylcyclopropyl)sulfonyl]-trans-(8CI) (NQ) and saquinavir can target main proteases through authoritative interaction to the catalytic dyad (Cys145 and His41) of SARS-CoV-2 3CLpro (Figures 1F,H), and along these lines are believed to hinder the protease activity, as has been reported with anti-SARS-CoV-2 agent (lopinavir). As referenced before, we identified the NQ from the methanolic leaf extract of *D. palmatus* using GC-MS analysis (Alexpandi et al., 2019). Unfortunately, the NQ is not available commercially. Henceforth, the present study essentially evaluated their drug-likeness, bioactivity, and ADMET properties through an *in silico* approach. The outcomes demonstrated that the NQ has low _mi_log*P* (3.63) and low TPSA value (37.38), which authenticates the ideal lipophilicity (_mi_log*P* < 5) nature and higher oral-absorption or membrane permeability (TPSA < 100) than other quinolines. These are the physicochemical properties that play a fundamental role in deciding the ADMET properties of compounds (Shen et al., 2012; Han et al., 2019). In ADMET analysis, the results reveal that the NQ was predicted as Caco-2+, HIA+, a non-inhibitor to P-glycoprotein, BBB+, a non-inhibitor to CYP2D6, a non-substrate to CYP3A4, and a non-inhibitor to excretion-related receptors such as OCT-1, OCT-2, and MATE-1. The NQ showing some toxic impact on the human either-a-go-go related genes, has even been predicted as a non-carcinogenic, non-mutagenic, non-eye irritant, and non-eye corrosive. These results unequivocally suggest that the novel phytocompound, quinoline,1,2,3,4-tetrahydro-1-[(2-phenylcyclopropyl)sulfonyl]-trans-(8CI), can be used as a protease-inhibitor drug for the treatment of COVID-19, but the compound needs to be synthesized.

Saquinavir, the first FDA-approved HIV-1 drug, has the ability to cleave between Tyr-Pro or Phe-Pro of the HIV polyproteins, which is rare in mammalian systems (Noble and Faulds, 1996). Hence, saquinavir does not interfere with mammalian proteases, symbolizing its safety level for humans (Ganguly et al., 2011). Saquinavir is a protease inhibitor that binds to the active site of the viral protease and thereby blocks cleavage of viral polyproteins and maturation of the HIV-1 and HIV-2 (Kim et al., 1998). Furthermore, (Tan et al., 2004) showed the *in vitro* antiviral activity of saquinavir against SARS-CoV-1. The present study revealed that saquinavir is able to bind with the catalytic dyad, and is thereby anticipated to interrupt 3CLpro activity (Figure 1H). So, we recommend saquinavir as the potent protease-inhibitor for drug-repurposing against COVID-19.

In the research of anti-SARS-CoV-2 drug designing, RdRp has been well-thought-out as an incredibly potent drug target due to its central role in RNA-synthesis from RNA-templates (Gao et al., 2020). In addition, RdRp-inhibitors do not show considerable toxicity or side effects on host cells (Dong et al., 2020). The active site of the SARS-CoV-2 RdRp domain is formed by the conserved polymerase motifs A-G, within 549th to 776th aminoacid residues, which are essential for the RNA-directed 5'-3' polymerase activity (Shannon et al., 2020). As in other RNA-polymerases, the template/primer entry (known as nucleoside triphosphate (NTP) entry) and budding strand are congregating in a central cavity where the RdRp motifs intercede RNA-template mediated RNA synthesis in SARS-CoV-2. The NTP entry channel of SARS-CoV-2 is placed in the set of hydrophilic residues, including Lys545, Arg553, and Arg555 in motif-F (Gao et al., 2020). Therefore, the nucleotide-analog antiviral inhibitors such as remdesivir and favipiravir also showed their antiviral potential against SARS-CoV-2 (Gordon et al., 2020). Our results revealed that both elvitegravir and oxolinic acid have the ability to bind with the NTP binding channel of SARS-CoV-2 with a low binding energy (−7.1 Kcal/mol) (Figures 4E,G), similar to the parental nucleotides such as ATP (−7.6 Kcal/mol), UTP (−7.1 Kcal/mol), GTP (−7.7 Kcal/mol), and CTP (−7.1 Kcal/mol). We believed that these two quinolines can more readily interact with the NTP binding channel than parental-nucleotides (especially than UTP and CTP), and thereby, possibly block the *de novo* addition of NTP to the 3'-OH strand, which leads to the arrest of viral replication (Gao et al., 2020), as illustrated in Figure S4. Elvitegravir is an integrase inhibitor used for the anti-retroviral treatment of HIV-1 (Shimura et al., 2008; Ramanathan et al., 2011). Oxolinic acid is a synthetic quinoline-derived antibiotic used to treat bacteria causing urinary tract infections (Sato et al., 2006; Irgi et al., 2015). Owing to the nucleotide-antagonistic behavior, we suggest that elvitegravir and oxolinic acid might be the potent RdRp inhibitors for SARS-CoV-2 in the treatment of COVID-19.

On the other hand, ACE2 is a type I transmembrane metallocarboxypeptidase, an enzyme that plays a crucial role in the rennin-angiotensin (RAS) system and is considered as a target for the treatment of hypertension (Burrell et al., 2004). ACE2 is widely distributed in the human body and has been associated with the protective function in the cardiovascular system and other organs (Yagil et al., 2003). In contrast, human ACE2 is the recognized functional receptor for the Spike glycoprotein of SARS-CoV-2 that initiates cell entry into host cells and viral replication in the target cells (Lan et al., 2020). The previous report proved that ACE2 knockout significantly reduces the viral load in mice after the experimental SARS-CoV infection (Hoffmann et al., 2020). As shown in Figure 3, though several quinolines could bind with ACE2, none was found to bind with the RBD of the ACE2-Spike complex. Moreover, these kinds of ACE2 inhibitors may not be appropriate for treating COVID-19 because these drugs can inhibit ACE2 enzyme activities, and cause lung injury and heart failure (Velkoska et al., 2016). To predict the RBD interface binding compound, we found one quinoline-drug, rilapladib, was targeting the RBD of the Spike-ACE2 complex, as shown in Figure 6B. Rilapladib, a hydroquinoline-based small molecule drug developed by GlaxoSmithKline was used as a lipoprotein-associated phospholipase A2 (Lp-PLA2) inhibitor or 1-alkyl-2-acetylglycerophosphocholine esterase inhibitor for treating atherosclerotic plaques and Alzheimer's disease (Shaddinger et al., 2014). It's worth mentioning that rilapladib was well-fitted into the interface of the RBD of the Spike-ACE2 complex (Figure 6B), in which lots of interactions with His34, Glu35, Glu37, Asp38, Leu39, Lys68, Ala71, Phe72, Glu75, Gln76, Leu79, and Lys353 create a strong binding with the interface of RBD and block the Spike-ACE2 interactions. Owing to the formation of possible interactions at the RBD interface of the Spike-ACE2 complex, the present study suggests rilapladib prevents ACE2-mediated viral entry of SARS-CoV-2.

Moreover, the *in silico* ADMET results demonstrated that these quinolines were non-toxic, non-carcinogenic, absorb in the human intestine, have Caco-2 permeability, do not inhibit CYP enzymes, are non-inhibitors for RCT, and non-inhibitors for Human Ether-a-go-go related genes, which suggested their significant pharmacokinetic properties. Further, the drug half-life of selected quinoline drugs are significantly long, in which these quinolines were expected to offer an efficient drug distribution against COVID-19. Overall, we believe that these quinolines may be efficient drug candidates for the development of efficient therapeutics against COVID-19 in this pandemic period.

## Conclusion

In conclusion, the current investigation recommends that the novel phyto-quinoline (quinoline,1,2,3,4-tetrahydro-1-[(2-phenylcyclopropyl)sulfonyl]-trans-(8CI)) and the existing quinoline-based drugs saquinavir, elvitegravir, and oxolinic acid could be used as potent inhibitors for SARS-CoV-2. Subsequently, rilapladib is suggested for anti-Spike-RBD-ACE2 therapy to avoid ACE2-mediated viral entry of SARS-CoV-2 into the host cells. Notwithstanding, further *in vitro* and *in vivo* experiments are needed to transform these potential inhibitors into clinical drugs. We anticipate that this new finding could significantly impact the development of therapeutic agents for COVID-19 in the future.

## Data Availability Statement

All datasets presented in this study are included in the article/Supplementary Material.

## Author Contributions

RA: conceptualization, performed the experiments, data analysis, and writing-original draft. JD: data analysis and reviewing-original draft. SP: supervision and reviewing-original draft. AR: conceptualization, supervision, and reviewing-original draft. All authors contributed to the article and approved the submitted version.

## Conflict of Interest

The authors declare that the research was conducted in the absence of any commercial or financial relationships that could be construed as a potential conflict of interest.
