# Supplementary material for: Quinolines-Based SARS-CoV-2 3CLpro and RdRp Inhibitors and Spike-RBD-ACE2 Inhibitor for Drug-Repurposing Against COVID-19: An in silico Analysis
Source: Front Microbiol. 2020 Jul 23;11:1796. doi: 10.3389/fmicb.2020.01796 (PMC7390959; doi:10.3389/fmicb.2020.01796)
Supplement: Supplementary file 1 [file Data_Sheet_1.PDF]

Supplementary Material

Quinolines-based SARS-CoV-2 3CLpro and RdRp inhibitors and Spike-RBD-ACE2 inhibitor for drug-repurposing against COVID-19: An *in silico* analysis

Rajaiah Alexpandi[1], Joelma Freire De Mesquita[2], Shunmugiah Karutha Pandian[1], and Arumugam Veera Ravi[1]*

[1]Department of Biotechnology, School of Biological Sciences, Science Campus, Alagappa University, Karaikudi-630 003, India.

[2]Laboratory of Bioinformatics and Computational Biology, Department of Genetics and Molecular Biology, Federal University of Rio de Janeiro State (UNIRIO), Rio de Janeiro, Brazil.

Corresponding Author:

Dr. A. Veera Ravi,

Professor,

Department of Biotechnology,

School of Biological Sciences,

Alagappa University,

Karaikudi- 630 003,

India.

E-mail: aveeraravi@rediffmail.com

Supplementary Figures

Fig. S1 (a) depicts the binding region of the reported anti-COVID-19 drug (Lopinavir) (-6.6 Kcal/mol) with SARS-CoV-2 3CLpro, respectively. (b) shows the super-position view of the binding sites and their interacting amino acids of lopinavir with SARS-CoV-2 3CLpro. (c) indicates the interaction with the active catalytic dyad of SARS-CoV-2 3CLpro (Cys145 and His41). (d) Hydrogen bond formation of lopinavir with Arg188 residues of 3CLpro at 1.824 Å distance. (e) reveal the interacted aminoacid residues of SARS-CoV-2 3CLpro with lopinavir.

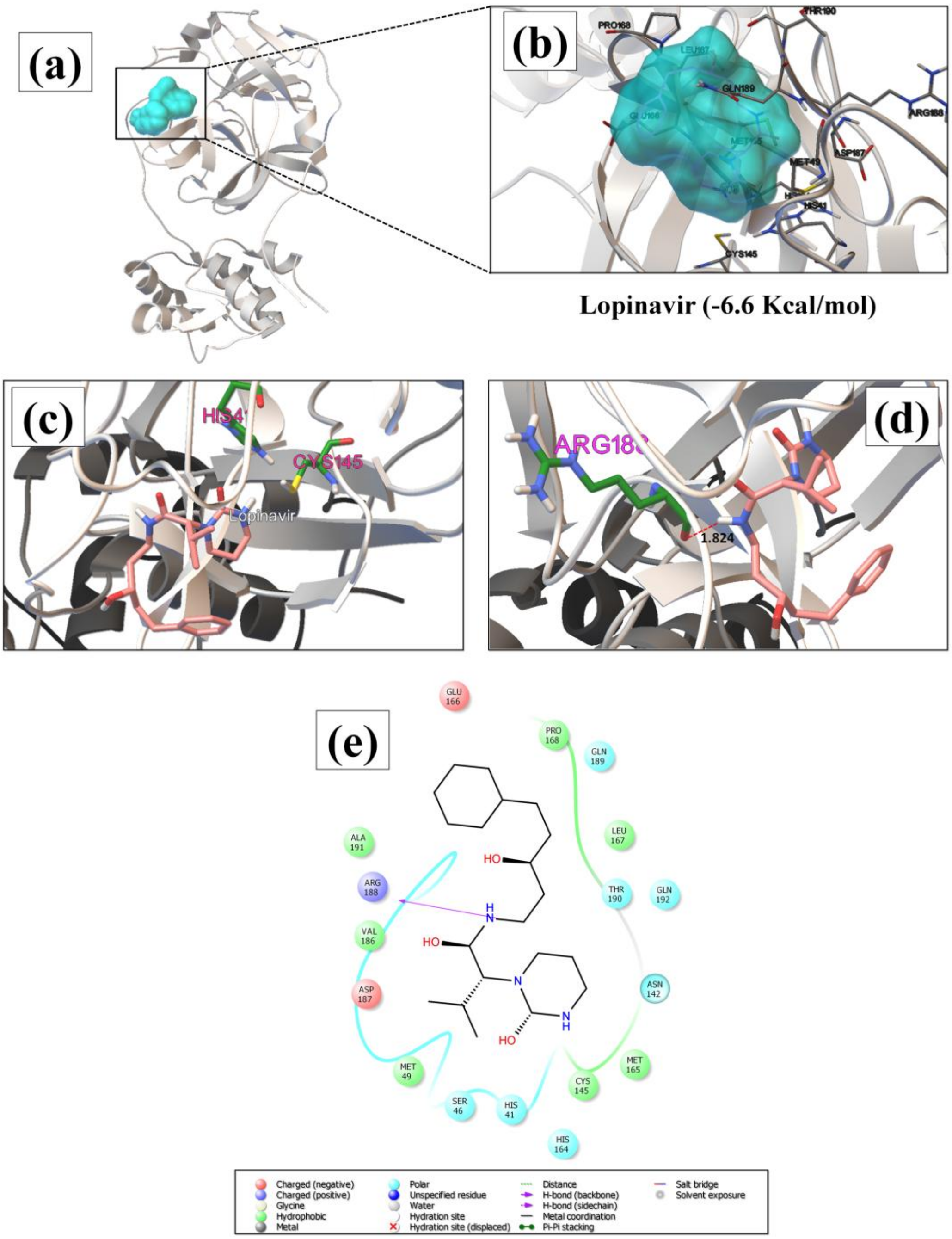

Fig. S2 Binding patterns with the NTP entry channel (a set of hydrophilic residues such as Lys545, Arg553, and Arg555) as well as their interacted aminoacid residues of SARS-CoV-2 RdRp by natural-nucleotides such as ATP (-7.6 Kcal/mol), UTP (-7.1 Kcal/mol), GTP (-7.7 Kcal/mol), and CTP (-7.1 Kcal/mol).

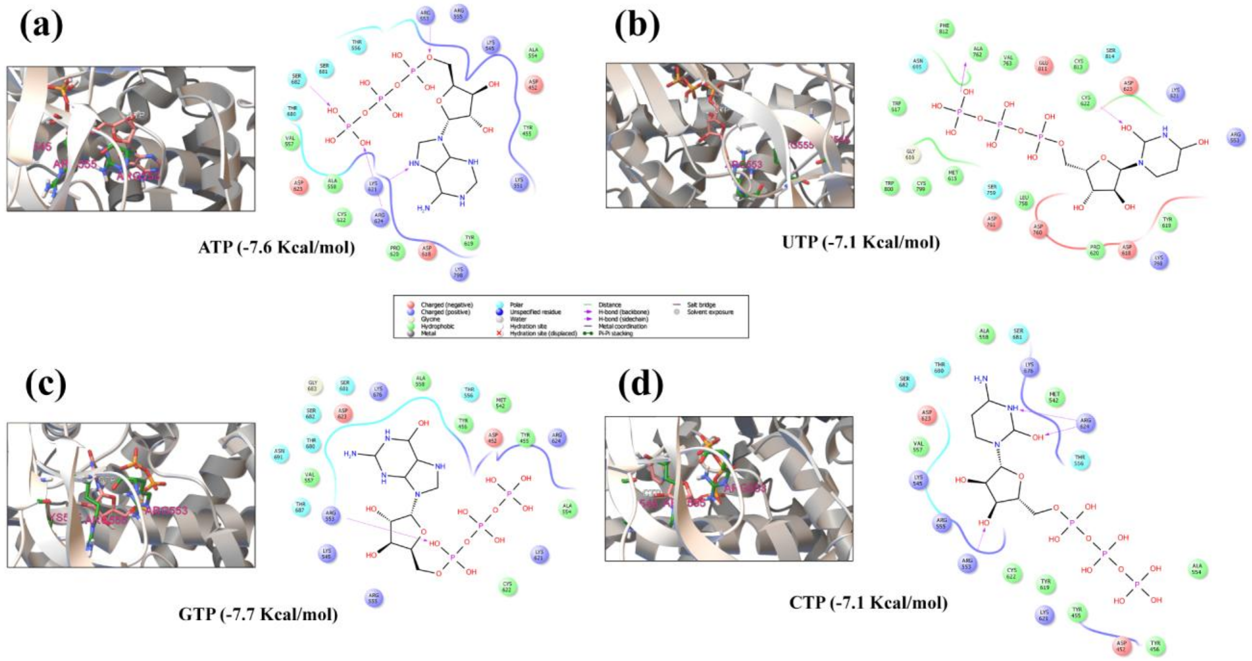

Fig. S3 (a) & (b) shows the binding region and close-up view of the interacting amino acids of the reported anti-COVID-19 drug (triphosphate form of remdesivir) (-7.8 Kcal/mol) with SARS-CoV-2 RdRp. (c) visualizes the interaction with the NTP entry channel of SARS-CoV-2 RdRp (a set of hydrophilic residues such as Lys545, Arg553, and Arg555) by remdesivir. (d) Hydrogen bond formation of remdesivir triphosphate with Lys551, Arg553, Lys621, and Lys798 residues of RdRp at 2.354, 2.502, 2.072, and 2.011 Å distance, respectively. (e) illustrate the interacted aminoacid residues of SARS-CoV-2 RdRp with remdesivir triphosphate.

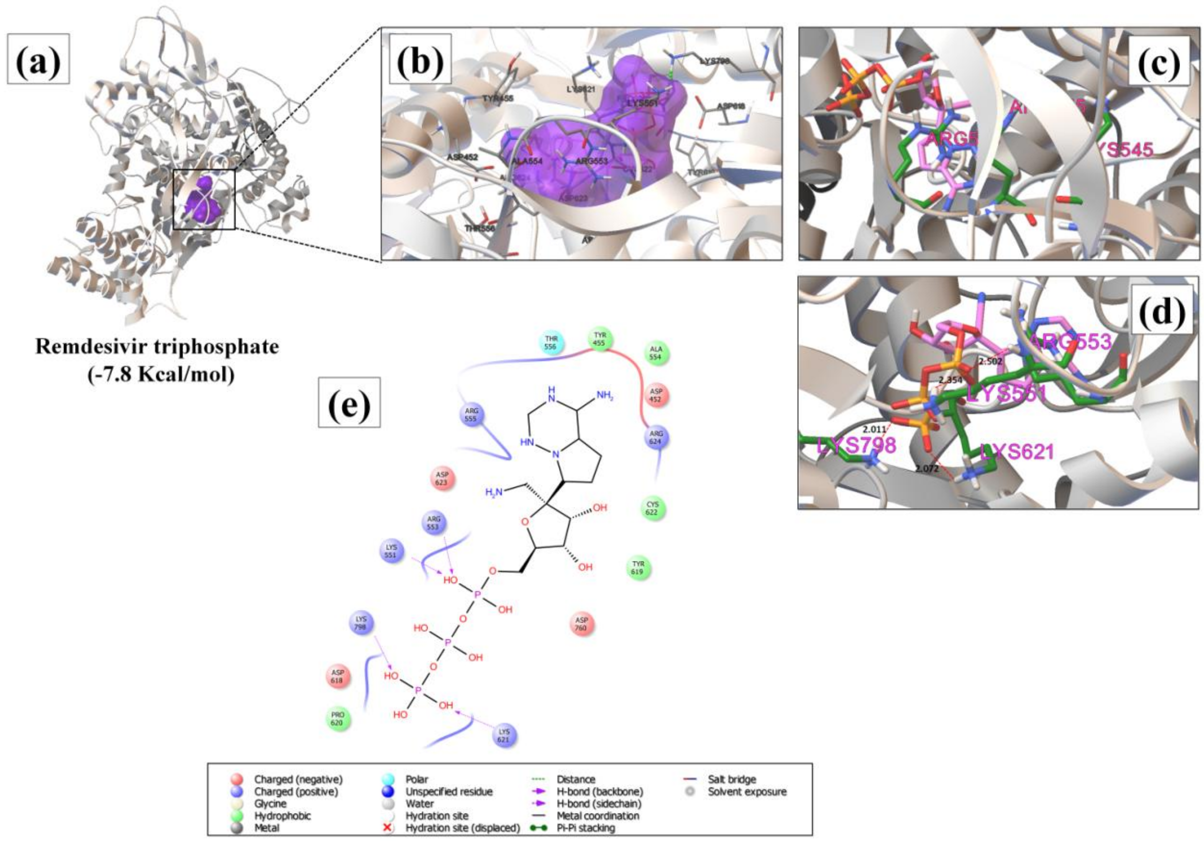

Fig. S4 Schematical representation of the inhibition mode of SARS-CoV-2 RdRp activity by elvitegravir and oxolinic acid.

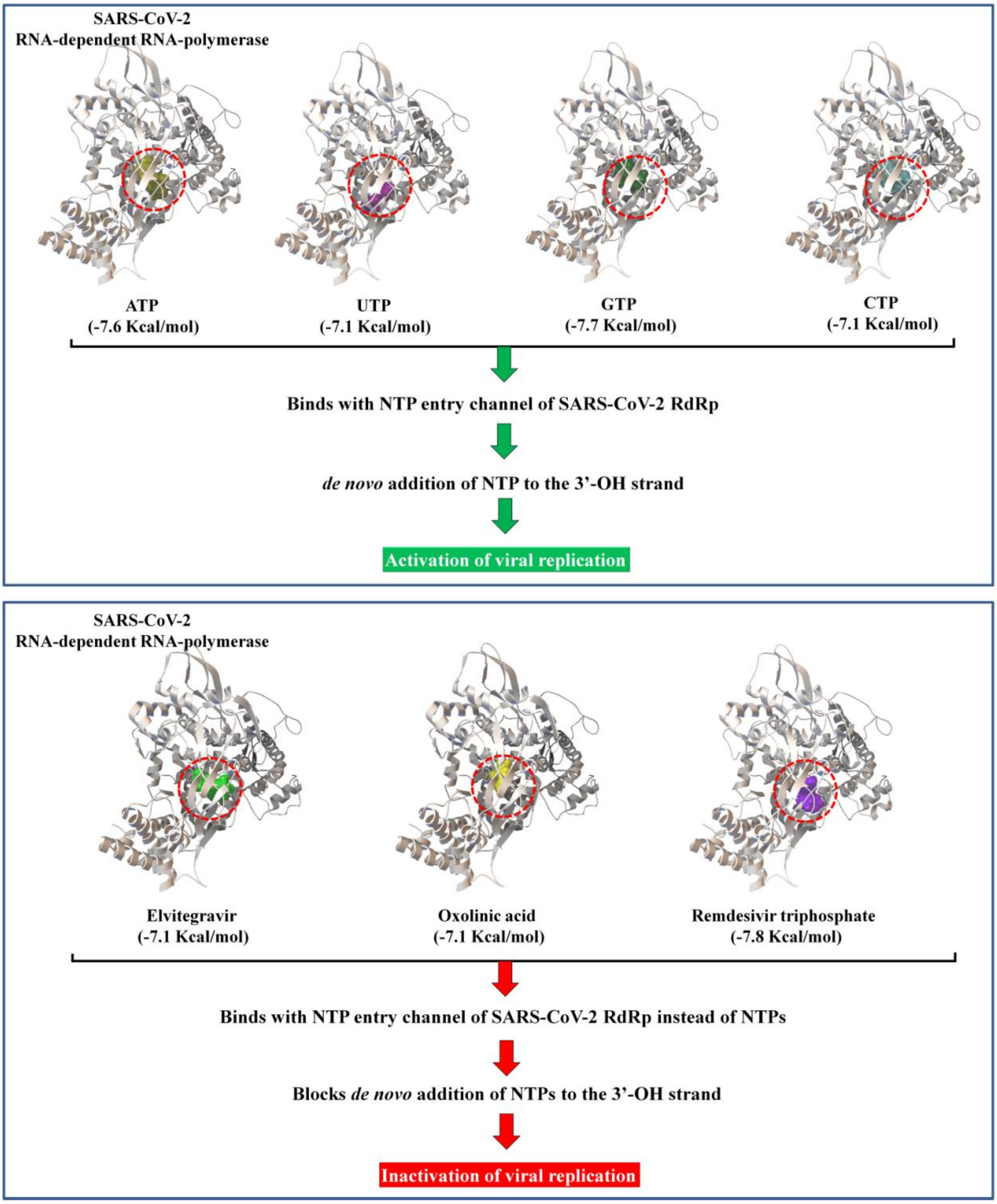

Fig. S5 shows the receptor binding domain (RBD) of SARS-CoV-2 Spike protein with human ACE-2 receptor with interacted aminoacid residues.

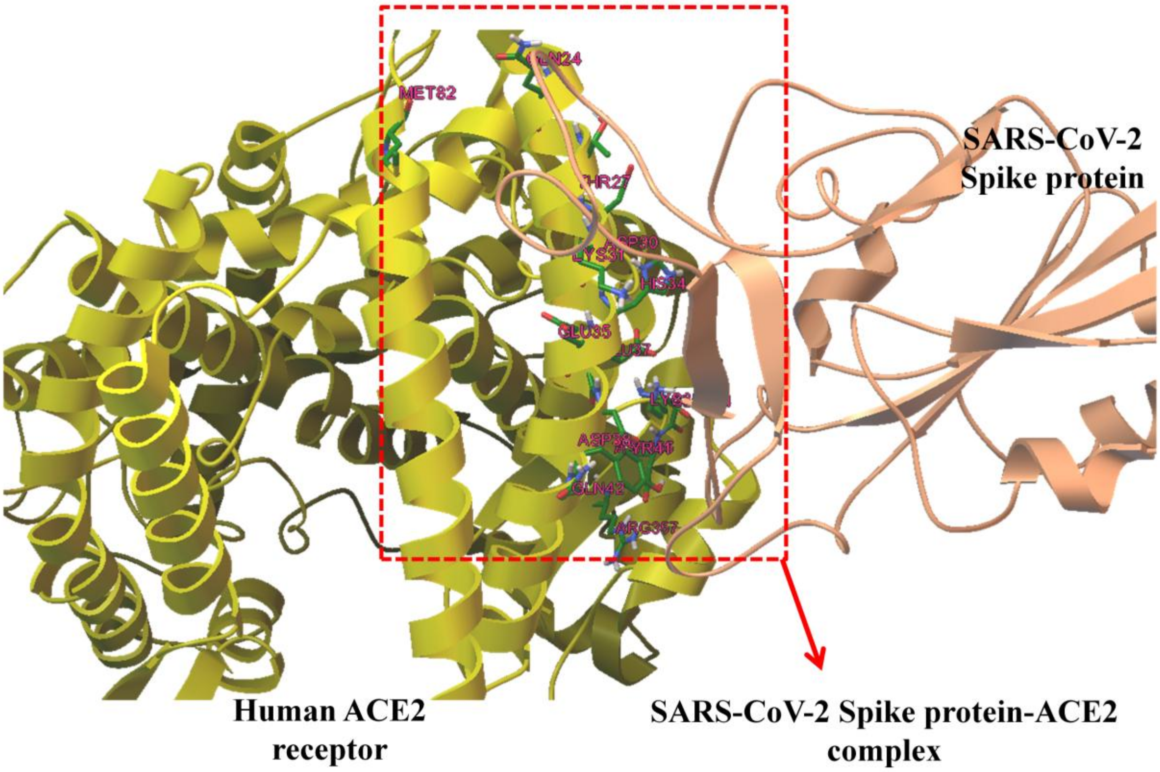

Fig. S6 *In silico* drug-likeness prediction of selected quinolines using the Molinspiration server.

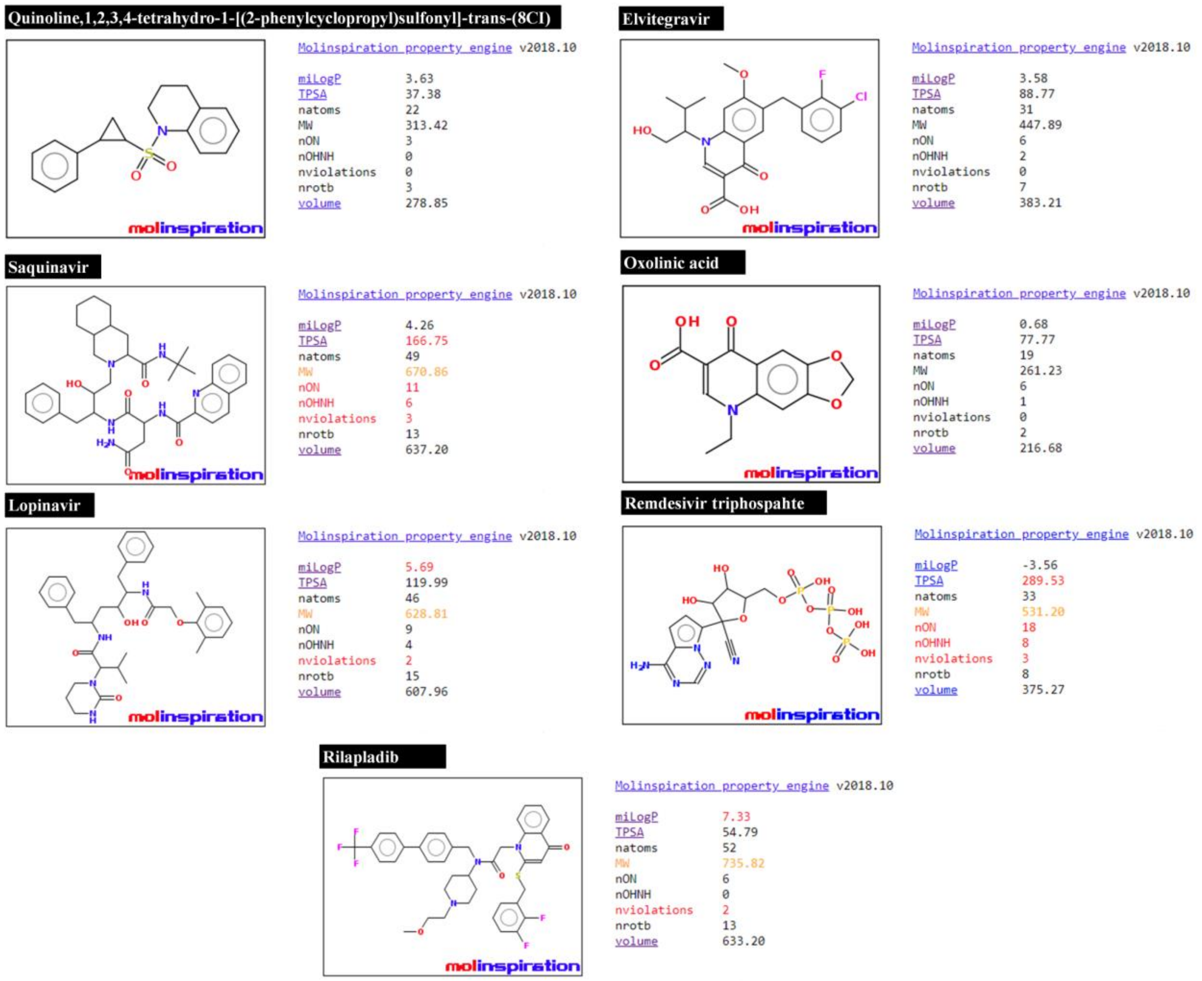

Fig. S7 *In silico* prediction of bioactivity score of the selected quinolines. The green-marking indicates the predicted bioactivity of the compounds by the Molinspiration server.

Quinoline,1,2,3,4-tetrahydro-1-[(2-phenylcyclopropyl)sulfonyl]-trans-(8CI)

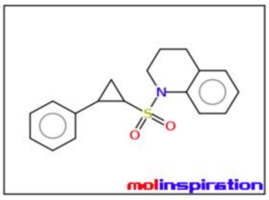

Molinspiration bioactivity score v2018.03	
GPCR ligand	0.16
Ion channel modulator	-0.06
Kinase inhibitor	-0.31
Nuclear receptor ligand	-0.08
Protease inhibitor	0.22
Enzyme inhibitor	-0.03

Get data as text (for copy / paste).

Get 3D geometry BETA

Elvitegravir

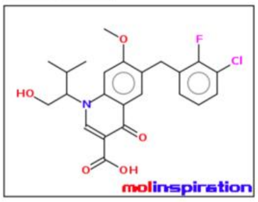

Molinspiration bioactivity score v2018.03	
GPCR ligand	0.08
Ion channel modulator	-0.25
Kinase inhibitor	-0.01
Nuclear receptor ligand	0.07
Protease inhibitor	-0.18
Enzyme inhibitor	0.38

Get data as text (for copy / paste).

Get 3D geometry BETA

Saquinavir

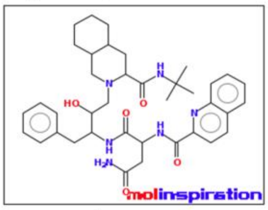

Molinspiration bioactivity score v2018.03	
GPCR ligand	-0.12
Ion channel modulator	-1.17
Kinase inhibitor	-0.87
Nuclear receptor ligand	-1.09
Protease inhibitor	0.40
Enzyme inhibitor	-0.61

Get data as text (for copy / paste).

Get 3D geometry BETA

Oxolinic acid

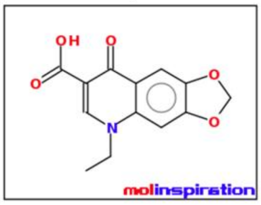

Molinspiration bioactivity score v2018.03	
GPCR ligand	-0.01
Ion channel modulator	-0.32
Kinase inhibitor	-0.22
Nuclear receptor ligand	-0.14
Protease inhibitor	-0.64
Enzyme inhibitor	0.21

Get data as text (for copy / paste).

Get 3D geometry BETA

Lopinavir

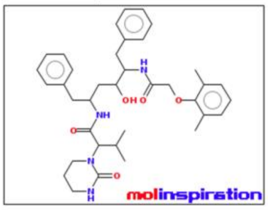

Molinspiration bioactivity score v2018.03	
GPCR ligand	0.04
Ion channel modulator	-0.78
Kinase inhibitor	-0.55
Nuclear receptor ligand	-0.66
Protease inhibitor	0.42
Enzyme inhibitor	-0.37

Get data as text (for copy / paste).

Get 3D geometry BETA

Remdesivir triphospahte

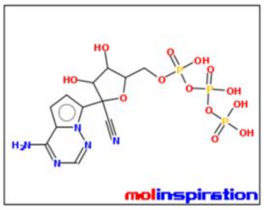

Molinspiration bioactivity score v2018.03	
GPCR ligand	1.06
Ion channel modulator	0.93
Kinase inhibitor	0.92
Nuclear receptor ligand	-0.11
Protease inhibitor	0.66
Enzyme inhibitor	1.16

Get data as text (for copy / paste).

Get 3D geometry BETA

Rilapladib

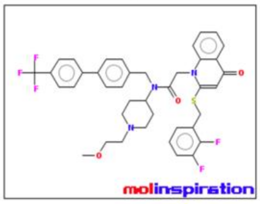

Molinspiration bioactivity score v2018.03	
GPCR ligand	-0.85
Ion channel modulator	-2.03
Kinase inhibitor	-1.42
Nuclear receptor ligand	-1.60
Protease inhibitor	-0.58
Enzyme inhibitor	-1.30

Get data as text (for copy / paste).

Get 3D geometry BETA

Supplementary Tables

Supplementary Table 1: iGEMDOCK-based virtual-screening of potent SARS-CoV-2 inhibitors from the phytochemicals of *Diplocyclos palmatus*.

Peak No.	PubChem CID	Name	Binding energy with 3CLpro	Binding energy with RdRp	Binding energy with ACE2
**1**	22833308	Bicyclo[3.1.1]heptane,2,6,6-trimethyl-, (1R,2S,5R)-rel-	-87.5	-82.4	-55.3
**2**	8181	Methyl hexadecanoate	-74.3	-88.2	-82.7
**3**	985	Palmitic acid	-81.5	-80.4	-88.2
**4**	5281	Stearic Acid	-82.5	-77.5	-88.7
**5**	5280590	Elaidic acid methyl ester	-81.5	-71.4	-86.8
**6**	5280435	Phytol	-89.2	-83.4	-91.7
**7**	3931	9,12-Octadecadienoicacid	-69.7	-88.1	-72.4
**8**	445639	Oleic acid	-81.1	-76.4	-77.8
**9**	11634	6-Octadecenoic acid	-88.5	-75.7	-84.9
**10**	NSC 140237 (Guidechem)	Quinoline,1,2,3,4-tetrahydro-1-[(2-phenylcyclopropyl)sulfonyl]-, trans- (8CI)	-136.4	-98.4	-102.1
**11**	14900	Glycerol 1-palmitate	-88.4	-73.7	87.4
**12**	CTK2E4706 (Chemtik ID)	Silane, [2-[dimethyl(3-methyl-3-butenyl)silyl]phenyl] dimethylphenyl-	---	---	---
**13**	33022	9-Octadecenoic acid(9Z)-, 2,3-dihydroxypropyl ester	-82.7	-81.7	-89.4
**14**	638072	Squalene	-91.5	-86.4	-92.0
**15**	5282350	Delta-Tocopherol	-89.5	-91.2	-98.8
**16**	6857447	Beta-Tocopherol	-94.4	-89.2	-97.5
**17**	92729	Gamma-Tocopherol	-92.1	-90.4	-106.5

Supplementary Table 2: iGEMDOCK-based virtual-screening of 3CLpro-inhibitors from the quinoline-drugs database.

PubChem ID	DrugBank Accession Number	Drug name	Binding energy (Kcal/mol)
441243	DB01232 (APRD00623)	Saquinavir	-204.2
9918381	DB05119 (DB05256)	Rilapladib	-186.3
4628	DB13627	Oxolinic acid	-126.5
5277135	DB09101 (DB05618)	Elvitegravir	-115.1
10372836	DB12526	Batefenterol	-112.7
461399	DB13261	Sitafloxacin	-111.7
347828848	DB12640	CP-609754	-107.9
9827968	DB12137	GSK-256066	-107.1
3086677	DB09335	Alatrofloxacin	-106.4
11635763	DB06638	Quarfloxin	-105.4
62959	DB00685 (APRD01281)	Trovafloxacin	-103.9
51039030	DB14993	Pyrotinib	-103.8
49831411	DB14185 (DBSALT001351)	Aripiprazole lauroxil	-103
11993740	DB06600	Nemonoxacin	-102.7
5388906	Bedaquiline	Bedaquiline	-102.1
487101	DB11943	Delafloxacin	-101.8
159324	DB04960	Tipifarnib	-101.8
56206	DB11511	Difloxacin	-100.5
135565932	DB12051	Setrobuvir	-100.2
57519748	DB12167	LY-3023414	-99.4
2754	DB01166 (APRD00155)	Cilostazol	-99.3
65947	DB11892	Prulifloxacin	-99
56208	DB11491	Sarafloxacin	-98.6
12004316	DB12211	PSI-697	-97.9
11962616	DB12100	Abediterol	-97.5
60063	DB14025	Clinafloxacin	-97.3
16220172	DB08820 (DB05989)	Ivacaftor	-97.1
5379	DB01044 (APRD00996)	Gatifloxacin	-97
3357	DB04576	Fleroxacin	-95.8
29112	DB11394	Decoquinate	-95.7
4046	DB00358 (APRD00300)	Mefloquine	-95.3
152946	DB00218 (APRD00281)	Moxifloxacin	-95.1

124093	DB06160	Garenoxacin	-95
9802884	DB11453	Pradofloxacin	-95
10178705	DB06771 (DB05862)	Besifloxacin	-94.9
72474	DB00365 (APRD01003)	Grepafloxacin	-94.9
71188	DB11404	Enrofloxacin	-94.8
135398510	DB05928	Dovitinib	-94.4
60605	DB11443	Orbifloxacin	-94.3
149096	DB01137 (APRD00477, DB06085)	Levofloxacin	-94.1
11567473	DB09047	Finafloxacin	-94
60021	DB01405	Temafloxacin	-93.2
6445562	DB05524	Pelitinib	-93
2764	DB00537 (APRD00424, EXPT00999)	Ciprofloxacin	-92.8
115358	DB06608	Tafenoquine	-92.5
4583	DB01165 (APRD00502)	Ofloxacin	-92.2
11978813	DB09128	Brexpiprazole	-91.3
23673836	DB13379	Chiniofon	-91.3
71335	DB11393	Danofloxacin	-91.3
6918554	DB05039	Indacaterol	-91.3
2165	DB00613 (APRD00796)	Amodiaquine	-90.8
4410	DB12447	Nadifloxacin	-90.2
9863827	DB12924	Ozenoxacin	-90.2
50294	DB00716 (APRD01137)	Nedocromil	-90.1
5042	DB11656	Rebamipide	-90.1
6432105	DB15300	Hydroquinidine	-89.5
9892540	DB05422	OPC-14523	-89.2
149096	DB01137 (APRD00477, DB06085)	Levofloxacin	-89.1
688561	DB01366	Procaterol	-89.1
11560224	DB08089	LGD-2226	-89
3649	DB13718	Hydroquinine	-87.9
9952872	DB12479	Zabofloxacin	-87.8
57469	DB00724 (APRD01030)	Imiquimod	-87.4
58258	DB13772	Rufloxacin	-86.8
54682876	DB05861	Tasquinimod	-86.8
3025	DB00527 (APRD00915)	Cinchocaine	-86.7

54676478	DB11366	Roquinimex	-86.6
3086401	DB09097	Quinagolide	-86.4
60795	DB01238 (APRD00638)	Aripiprazole	-86.3
2993	DB04209 (EXPT01162, DB11615)	Dequalinium	-86.3
16750192	DB05234	LGD2941	-86.3
2583	DB00521 (APRD00195)	Carteolol	-85.9
26383	DB11433	Nequinate	-85.9
65957	DB11774	Pazufloxacin	-85.8
51081	DB00487 (APRD00108)	Pefloxacin	-85.8
60651	DB11426	Marbofloxacin	-85.4
9571107	DB01155 (APRD00053)	Gemifloxacin	-85.3
60464	DB01208 (APRD01231	Sparfloxacin	-85.3
4612	DB01096 (APRD01150)	Oxamniquine	-84.8
10239	DB13596	Oxycinchophen	-84.7
21651	DB11378	Buquinolate	-84.3
4539	DB01059 (APRD00469)	Norfloxacin	-84.1
3374	DB08972	Flumequine	-84
287180	DB00817 (APRD00369)	Rosoxacin	-83.1
3229	DB00467 (APRD00947)	Enoxacin	-83
54677946	DB06685	Laquinimod	-83
25195253	DB13937	LGD-3303	-82.9
2719	DB00608 (APRD00468)	Chloroquine	-82.2
1065	DB00468 (APRD00563)	Quinine	-81.7
441074	DB00908 (APRD00136)	Quinidine	-81.6
4421	DB00779 (APRD01133)	Nalidixic acid	-81
5281035	DB06816	Pyrvinium	-80.5
5273394	DB08278	1-(2-cyclopropylethyl)-3-(1,1-dioxo-2H-1,2,4-benzothiadiazin-3-yl)-6-fluoro-4-hydroxy-2(1H)-quinolinone	-78.3
604519	DB13668	Ipidacrine	-77.2
42548	DB04909	Sitamaquine	-76.6
3652	DB01611	Hydroxychloroquine	-76.5
1561	DB07918	2-heptyl-4-hydroxyquinoline N-oxide	-76
1779	DB01931	5,7-Dichlorokynurenic acid	-75

	(EXPT01212)		
124075	FDB011377 (primary ID)	Casimiroin	-74.5
6301	DB13306	Chlorquinaldol	-73.5
19910	DB01422	Nitroxoline	-73.2
4908	DB01087 (APRD00604)	Primaquine	-72.5
2453	DB13536	Broxyquinoline	-71.1
3728	DB09115	Diiodohydroxyquinoline	-71.1
2788	DB04815	Clioquinol	-70.8
2722	DB01243 (APRD00866)	Chloroxine	-70.7
3845	DB11937	Kynurenic Acid	-66.5
65592	DB13222	Tilbroquinol	-65.3
517000	DB11145	Oxyquinoline	-63.7
6038	DB04745	2-Hydroxyquinoline	-62.5
23649245	DB11692	Pavinetant	-41.2

Supplementary Table 3: iGEMDOCK-based virtual-screening of RdRp-inhibitors from the quinoline-drugs database.

PubChem ID	DrugBank Accession Number	Drug Name	Binding energy (Kcal/mol)
5277135	DB09101 (DB05618)	Elvitegravir	-323.4
4628	DB13627	Oxolinic acid	-277.2
441243	DB01232 (APRD00623)	Saquinavir	-126
124093	DB06160	Garenoxacin	-124.1
9918381	DB05119 (DB05256)	Rilapladib	-119
6445562	DB05524	Pelitinib	-118.3
56206	DB11511	Difloxacin	-112.1
10372836	DB12526	Batefenterol	-109.2
71335	DB11393	Danofloxacin	-107.6
11560224	DB08089	LGD-2226	-105.9
60605	DB11443	Orbifloxacin	-104.2
51039030	DB14993	Pyrotinib	-104.2
3086677	DB09335	Alatrofloxacin	-104.1
135565932	DB12051	Setrobuvir	-104
71188	DB11404	Enrofloxacin	-103.4
16220172	DB08820 (DB05989)	Ivacaftor	-103.4
347828848	DB12640	CP-609754	-102.6
60464	DB01208 (APRD01231	Sparfloxacin	-102.5
6918554	DB05039	Indacaterol	-99.9
9827968	DB12137	GSK-256066	-99.2
2764	DB00537 (APRD00424, EXPT00999)	Ciprofloxacin	-98.8
115358	DB06608	Tafenoquine	-98.8
149096	DB01137 (APRD00477, DB06085)	Levofloxacin	-98.1
461399	DB13261	Sitafloxacin	-98
16750192	DB05234	LGD2941	-96.6
57519748	DB12167	LY-3023414	-96.6
5388906	Bedaquiline	Bedaquiline	-96.4
26383	DB11433	Nequinate	-96
11635763	DB06638	Quarfloxin	-95.7
10178705	DB06771 (DB05862)	Besifloxacin	-95.5
51081	DB00487 (APRD00108)	Pefloxacin	-95.3

60063	DB14025	Clinafloxacin	-95.2
2583	DB00521 (APRD00195)	Carteolol	-94.8
3649	DB13718	Hydroquinine	-94.6
65947	DB11892	Prulifloxacin	-94.2
135398510	DB05928	Dovitinib	-93.5
60795	DB01238 (APRD00638)	Aripiprazole	-92.7
11962616	DB12100	Abediterol	-92.1
688561	DB01366	Procaterol	-91.9
29112	DB11394	Decoquinate	-91.7
58258	DB13772	Rufloxacin	-91.6
11993740	DB06600	Nemonoxacin	-91.2
3357	DB04576	Fleroxacin	-89.9
50294	DB00716 (APRD01137)	Nedocromil	-89.9
9571107	DB01155 (APRD00053)	Gemifloxacin	-89.4
4410	DB12447	Nadifloxacin	-88.3
2719	DB00608 (APRD00468)	Chloroquine	-88.2
487101	DB11943	Delafloxacin	-88.2
4612	DB01096 (APRD01150)	Oxamniquine	-88.2
62959	DB00685 (APRD01281)	Trovafloxacin	-87.7
4583	DB01165 (APRD00502)	Ofloxacin	-87.6
159324	DB04960	Tipifarnib	-87.6
287180	DB00817 (APRD00369)	Rosoxacin	-87.4
6432105	DB15300	Hydroquinidine	-87
10239	DB13596	Oxycinchophen	-86.3
2754	DB01166 (APRD00155)	Cilostazol	-86.2
5379	DB01044 (APRD00996)	Gatifloxacin	-86.2
3374	DB08972	Flumequine	-85.6
3652	DB01611	Hydroxychloroquine	-85.6
56208	DB11491	Sarafloxacin	-85.6
19910	DB01422	Nitroxoline	-85.5
152946	DB00218 (APRD00281)	Moxifloxacin	-85.4
5273394	DB08278	1-(2-cyclopropylethyl)-3-(1,1-dioxo-2H-1,2,4-benzothiadiazin-3-yl)-6-fluoro-4-hydroxy-2(1H)-quinolinone	-84.5
60651	DB11426	Marbofloxacin	-83.9
11567473	DB09047	Finafloxacin	-83.6

5042	DB11656	Rebamipide	-83.5
3229	DB00467 (APRD00947)	Enoxacin	-83.4
9952872	DB12479	Zabofloxacin	-83.4
2993	DB04209 (EXPT01162, DB11615)	Dequalinium	-83.3
11978813	DB09128	Brexpiprazole	-83.2
12004316	DB12211	PSI-697	-83.2
23673836	DB13379	Chiniofon	-82.7
1561	DB07918	2-heptyl-4-hydroxyquinoline N-oxide	-82.3
3025	DB00527 (APRD00915)	Cinchocaine	-81.8
9863827	DB12924	Ozenoxacin	-81.7
42548	DB04909	Sitamaquine	-81.7
4421	DB00779 (APRD01133)	Nalidixic acid	-81.2
54677946	DB06685	Laquinimod	-80.4
5281035	DB06816	Pyrvinium	-80.4
54676478	DB11366	Roquinimex	-80.2
49831411	DB14185 (DBSALT001 351)	Aripiprazole lauroxil	-79.7
3086401	DB09097	Quinagolide	-78.9
4908	DB01087 (APRD00604)	Primaquine	-78.6
54682876	DB05861	Tasquinimod	-78.6
4539	DB01059 (APRD00469)	Norfloxacin	-77.6
3845	DB11937	Kynurenic Acid	-77.4
57469	DB00724 (APRD01030)	Imiquimod	-77
149096	DB01137 (APRD00477, DB06085)	Levofloxacin	-76.4
21651	DB11378	Buquinolate	-75.5
4046	DB00358 (APRD00300)	Mefloquine	-75.3
72474	DB00365 (APRD01003)	Grepafloxacin	-74.8
9802884	DB11453	Pradofloxacin	-74.8
604519	DB13668	Ipidacrine	-74.7
1065	DB00468 (APRD00563)	Quinine	-74.2
2165	DB00613 (APRD00796)	Amodiaquine	-74.1
441074	DB00908 (APRD00136)	Quinidine	-74.1
1779	DB01931	5,7-Dichlorokynurenic acid	-73.8

	(EXPT01212)		
9892540	DB05422	OPC-14523	-73.1
124075	FDB011377 (primary ID)	Casimiroin	-71.9
2453	DB13536	Broxyquinoline	-71.8
2788	DB04815	Clioquinol	-71.8
25195253	DB13937	LGD-3303	-71.8
65957	DB11774	Pazufloxacin	-71.3
6301	DB13306	Chlorquinaldol	-71.1
2722	DB01243 (APRD00866)	Chloroxine	-70.8
65592	DB13222	Tilbroquinol	-70.4
3728	DB09115	Diiodohydroxyquinoline	-69.5
517000	DB11145	Oxyquinoline	-69.5
60021	DB01405	Temafloxacin	-66.8
6038	DB04745	2-Hydroxyquinoline	-66.2
23649245	DB11692	Pavinetant	17.2

Supplementary Table 4: iGEMDOCK-based **v**irtual-screening of ACE2-inhibitors from the quinoline-drugs database.

PubChem ID	DrugBank Accession Number	Drug Name	Binding energy (Kcal/mol)
347828848	DB12640	CP-609754	-229.1
441243	DB01232 (APRD00623)	Saquinavir	-222.4
9918381	DB05119 (DB05256)	Rilapladib	-209.9
11635763	DB06638	Quarfloxin	-182
10372836	DB12526	Batefenterol	-162.6
4628	DB13627	Oxolinic acid	-155.8
3086677	DB09335	Alatrofloxacin	-146.5
135398510	DB05928	Dovitinib	-144.9
9827968	DB12137	GSK-256066	-138.2
5042	DB11656	Rebamipide	-134
5277135	DB09101 (DB05618)	Elvitegravir	-125.1
11567473	DB09047	Finafloxacin	-115.3
487101	DB11943	Delafloxacin	-112.7
9802884	DB11453	Pradofloxacin	-112.1
6432105	DB15300	Hydroquinidine	-112
3649	DB13718	Hydroquinine	-112
135565932	DB12051	Setrobuvir	-110.2
152946	DB00218 (APRD00281)	Moxifloxacin	-109.1
2993	DB04209 (EXPT01162, DB11615)	Dequalinium	-108.5
56206	DB11511	Difloxacin	-108.5
62959	DB00685 (APRD01281)	Trovafloxacin	-108.5
5379	DB01044 (APRD00996)	Gatifloxacin	-107.6
11962616	DB12100	Abediterol	-107.2
2754	DB01166 (APRD00155)	Cilostazol	-106.7
60021	DB01405	Temafloxacin	-106.2
21651	DB11378	Buquinolate	-105.8
124093	DB06160	Garenoxacin	-105.5
149096	DB01137 (APRD00477, DB06085)	Levofloxacin	-105.1
56208	DB11491	Sarafloxacin	-105
3357	DB04576	Fleroxacin	-104.4

65947	DB11892	Prulifloxacin	-104.1
6918554	DB05039	Indacaterol	-104
11993740	DB06600	Nemonoxacin	-103.8
23673836	DB13379	Chiniofon	-103.3
71335	DB11393	Danofloxacin	-103.1
5388906	Bedaquiline	Bedaquiline	-102.8
4410	DB12447	Nadifloxacin	-102.6
54682876	DB05861	Tasquinimod	-102.5
49831411	DB14185 (DBSALT001351)	Aripiprazole lauroxil	-102.4
60063	DB14025	Clinafloxacin	-102.3
60651	DB11426	Marbofloxacin	-102
60464	DB01208 (APRD01231	Sparfloxacin	-101.9
16220172	DB08820 (DB05989)	Ivacaftor	-101.8
16750192	DB05234	LGD2941	-101.5
6445562	DB05524	Pelitinib	-101.3
3086401	DB09097	Quinagolide	-101.3
51039030	DB14993	Pyrotinib	-101.1
10178705	DB06771 (DB05862)	Besifloxacin	-101
71188	DB11404	Enrofloxacin	-100.9
10239	DB13596	Oxycinchophen	-100.8
461399	DB13261	Sitafloxacin	-100.8
58258	DB13772	Rufloxacin	-99.9
12004316	DB12211	PSI-697	-99.8
1065	DB00468 (APRD00563)	Quinine	-99.8
159324	DB04960	Tipifarnib	-99.7
115358	DB06608	Tafenoquine	-99.4
2764	DB00537 (APRD00424, EXPT00999)	Ciprofloxacin	-99.2
149096	DB01137 (APRD00477, DB06085)	Levofloxacin	-99.1
4583	DB01165 (APRD00502)	Ofloxacin	-99
60605	DB11443	Orbifloxacin	-98.5
9863827	DB12924	Ozenoxacin	-98.3
2583	DB00521 (APRD00195)	Carteolol	-98
29112	DB11394	Decoquinate	-97.7
65957	DB11774	Pazufloxacin	-97.5
50294	DB00716	Nedocromil	-97.3

	(APRD01137)		
11560224	DB08089	LGD-2226	-97.2
9952872	DB12479	Zabofloxacin	-97
25195253	DB13937	LGD-3303	-95.7
11978813	DB09128	Brexpiprazole	-95.1
2165	DB00613 (APRD00796)	Amodiaquine	-94.8
287180	DB00817 (APRD00369)	Rosoxacin	-94
3229	DB00467 (APRD00947)	Enoxacin	-93
51081	DB00487 (APRD00108)	Pefloxacin	-92.7
3652	DB01611	Hydroxychloroquine	-92.5
72474	DB00365 (APRD01003)	Grepafloxacin	-92.2
5281035	DB06816	Pyrvinium	-92.2
54677946	DB06685	Laquinimod	-92
3374	DB08972	Flumequine	-91.8
4046	DB00358 (APRD00300)	Mefloquine	-91.4
57519748	DB12167	LY-3023414	-90.8
26383	DB11433	Nequinate	-90.8
60795	DB01238 (APRD00638)	Aripiprazole	-90.7
4539	DB01059 (APRD00469)	Norfloxacin	-89.9
688561	DB01366	Procaterol	-89.4
4612	DB01096 (APRD01150)	Oxamniquine	-89
54676478	DB11366	Roquinimex	-88.3
4908	DB01087 (APRD00604)	Primaquine	-88.1
9892540	DB05422	OPC-14523	-87.5
4421	DB00779 (APRD01133)	Nalidixic acid	-86.4
42548	DB04909	Sitamaquine	-85.9
57469	DB00724 (APRD01030)	Imiquimod	-85.6
441074	DB00908 (APRD00136)	Quinidine	-85.5
3025	DB00527 (APRD00915)	Cinchocaine	-85.2
9571107	DB01155 (APRD00053)	Gemifloxacin	-85.1
2719	DB00608 (APRD00468)	Chloroquine	-83.2
1561	DB07918	2-heptyl-4-hydroxyquinoline N-oxide	-82.7
5273394	DB08278	1-(2-cyclopropylethyl)-3-(1,1-dioxo-2H-1,2,4-	-82.2

		benzothiadiazin-3-yl)-6-fluoro-4-hydroxy-2(1H)-quinolinone	
1779	DB01931 (EXPT01212)	5,7-Dichlorokynurenic acid	-79.7
124075	FDB011377 (primary ID)	Casimiroin	-78.4
604519	DB13668	Ipidacrine	-72.4
3845	DB11937	Kynurenic Acid	-71.4
6038	DB04745	2-Hydroxyquinoline	-69.4
6301	DB13306	Chlorquinaldol	-68.5
3728	DB09115	Diiodohydroxyquinoline	-68.5
65592	DB13222	Tilbroquinol	-68.5
2453	DB13536	Broxyquinoline	-68.4
19910	DB01422	Nitroxoline	-67.6
2788	DB04815	Clioquinol	-66.9
2722	DB01243 (APRD00866)	Chloroxine	-64.4
517000	DB11145	Oxyquinoline	-62
23649245	DB11692	Pavinetant	125.1
